# Delocalized Lithium Ion Flux by Solid-State Electrolyte Composites Coupled with 3D Porous Nanostructures for Highly Stable Lithium Metal Batteries

**DOI:** 10.1021/acsnano.3c04526

**Published:** 2023-07-29

**Authors:** Jooyoung Lee, Hyunji Park, Jieun Hwang, Juran Noh, Choongho Yu

**Affiliations:** ^†^Department of Mechanical Engineering and ^‡^Department of Material Science and Engineering, Texas A&M University, College Station, Texas 77843, United States

**Keywords:** lithium metal, solid-state electrolyte, composite, carbon nanotube, delocalized lithium ion

## Abstract

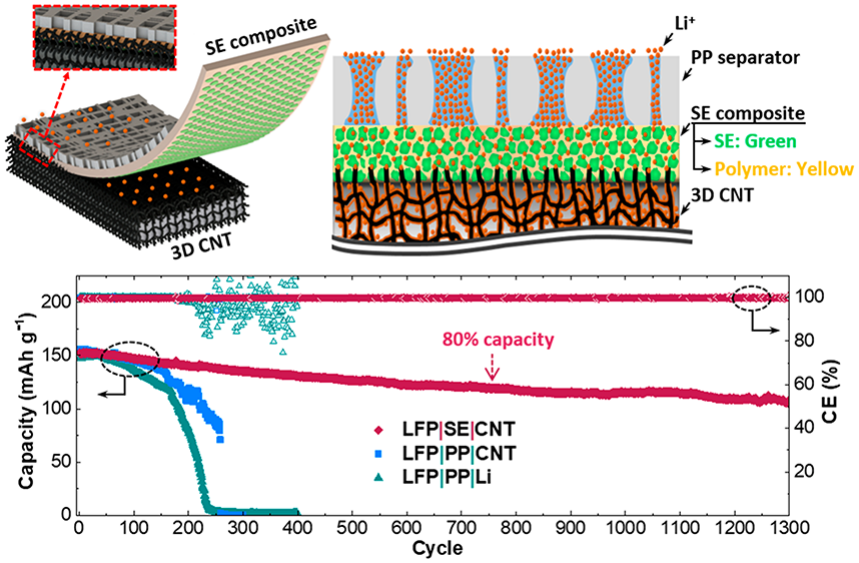

This work investigates the root cause of failure with the ultimate anode, Li metal, when employing conventional/composite separators and/or porous anodes. Then a feasible route of utilizing Li metal is presented. Our operando and microscopy studies have unveiled that Li^+^ flux passing through the conventional separator is not uniform, resulting in preferential Li plating/stripping. Porous anodes alone are subject to clogging with moderate- or high-loading cathodes. Here we discovered it is necessary to seek synergy from our separator and anode pair to deliver delocalized Li^+^ to the anode and then uniformly plate Li metal over the large surface areas of the porous anode. Our polymer composite separator containing a solid-state electrolyte (SE) can provide numerous Li^+^ passages through the percolated SE and pore networks. Our finite element analysis and comparative tests disclosed the synergy between the homogeneous Li^+^ flux and current density reduction on the anode. Our composite separators have induced compact and uniform Li plating with robust inorganic-rich solid electrolyte interphase layers. The porous anode decreased the nucleation overpotential and interfacial contact impedance during Li plating. Full cell tests with LiFePO_4_ and Li[Ni_0.8_Mn_0.1_Co_0.1_]O_2_ (NMC811) exhibited remarkable cycling behaviors: ∼80% capacity retention at the 750th and 235th cycle, respectively. A high-loading NMC811 (4 mAh cm^–2^) full cell displayed maximum cell-level energy densities of 334 Wh kg^–1^ and 783 Wh L^–1^. This work proposes a solution for raising energy density by adopting Li metal, which could be a viable option considering only incremental advancement in conventional cathodes lately.

## Introduction

The prosperity of electric vehicles demands high-energy-density batteries, which has substantially improved the cathodes of rechargeable lithium-based batteries over the past decades.^1,2^ Further increase of the energy density necessitates significant advances in their anodes. Lithium (Li) metal is the front runner because of its high specific capacity (3860 mAh g^–1^) compared to graphite (350 mAh g^–1^), the lowest electrode potential (−3.04 V vs SHE) over all possible alternatives, and a low gravimetric density (0.534 g cm^–3^).^3,4^ However, Li metal anodes have yet to be adopted in industry due to safety issues and rapid capacity fading. These detrimental side effects were caused primarily by Li dendrite growth as a result of nonuniform Li plating and stripping and large volume changes.^5−7^ The Li dendrite growth is considerably increasesd when the lithium-ion (Li^+^) flux is nonuniformly distributed over limited anode surfaces such as foil-like two-dimensional (2D) Li metal. The relatively large pore size and low porosity of conventional separators in typical liquid-electrolyte-based batteries make the Li^+^ flux concentrated on the anode. The limited surface areas of the 2D Li metal anode accompany large volume changes, which cause frequent disruption of solid-electrolyte interphase (SEI) layers, resulting in Li consumption and dendrite growth. To overcome these problems, separator-free all-solid-state batteries have been rigorously studied, but low ionic conductivity, narrow voltage windows, and unstable interfacial contacts are still challenging despite recent massive investments.^8−11^

Here the root cause of failure has been identified particularly when a pair of the conventional separator and Li metal foil, recently emerging composite separators, or porous anodes were used. Then an immediately deployable solution was suggested by exploiting the advantages of liquid and solid-state electrolytes, which do not require modifications in the cathode and operating conditions of conventional Li-ion batteries. More specifically, our study has sought synergistic effects by pairing composite separators containing a solid-state electrolyte (SE) and a 3D porous scaffold anode made of carbon nanotubes (CNTs) to mitigate the aforementioned problems. Recently, composite membranes have been examined as alternatives for conventional separators, mainly targeting the extended lifetime of Li-metal-based batteries.^12−16^ Nonetheless, the composites have shown limitations such as complicated manufacturing processes,^13,15^ undesirably thick composite layers,^15,16^ and poor capacity retention^13,14,16^ compared to typical polymer separators in conventional cells. In advanced Li-ion batteries coupling 2D Li metal anodes and high-capacity cathodes, Li dendrites were inevitably generated regardless of the distribution of Li^+^ flux near the separator due to the repetitive destruction and creation of the SEI during cycling, suggesting the importance of alleviating the local current density near the anode surface.

An effective approach for mitigating the limited surface of flat Li metal is to employ a 3D host framework as an anode.^17−22^ Porous frameworks are often made of electronically conducting materials, including porous copper, CNT, and graphene. The large surface areas of the frameworks can reduce the local current density and mitigate repeated breakage of the SEI layer by shrinking the volume change of the Li metal during stripping and plating. Unlike conventional graphite-based anodes, these cells are continuously charged beyond lithiation until the pores are filled with Li metal. However, the electric field near the inlet of the pores (i.e., separator side) of the electrode is stronger than the inner part, accelerating the preferential deposition of Li metal and eventually clogging the pore at the inlet (i.e., impairing the inner pores).^23,24^ To circumvent the clogging problem, the electric field at the pore inlet must be attenuated. Recent studies have shown that thin electronically insulating coating layers such as metal–organic framework^25^ and alumina layer^26^ are effective, but they require significant efforts to keep the layer thin enough to pass Li^+^ without considerably introducing unwanted impedance. Still, when Li^+^ flux is localized on this type of porous framework, Li dendrites tend to sprout from the surfaces of the porous anodes.

**Figure 1:**
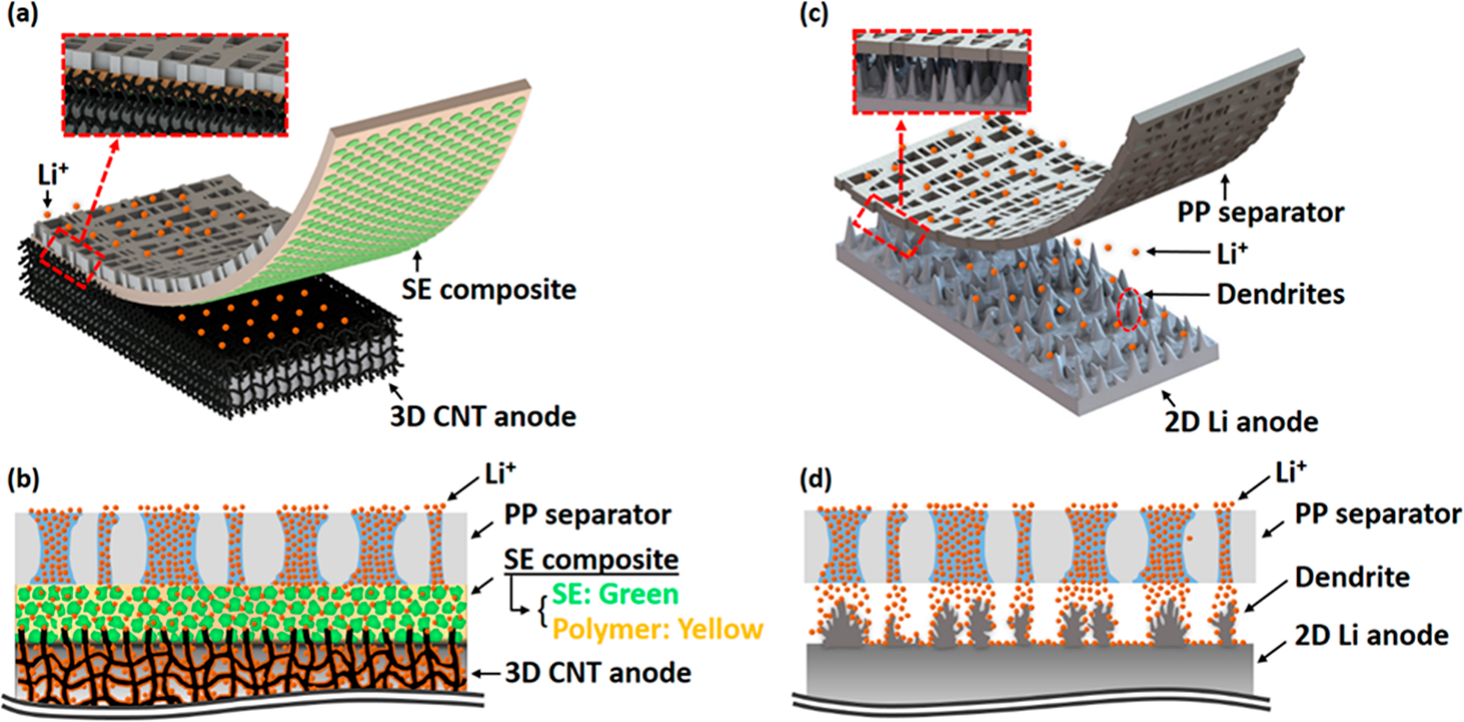
(a) Our solid-state electrolyte (SE) composite separator paired with a 3D CNT anode. The inset represents that the SE composite separator and 3D CNT framework are firmly attached by the large contact surfaces between the entangled CNT and polymer. (b) Cross-section displaying that the concentrated Li^+^ flux (orange dots) in a PP separator can be delocalized in the composite containing SE particles (green circles) and then inserted into the pores of the CNT framework (black lines) and plated on the CNT surface. The pores in the framework are filled with Li metal. (c) In the conventional PP separator and 2D Li metal anode pair, Li dendrites readily sprout from the Li metal anode toward the gap in between. (d) The nonuniformly distributed Li^+^ flux through the PP separator generates dendrites due to localized Li plating on the limited surface areas of 2D Li metal.

This work has demonstrated our synergistic approach coupling a composite separator and a porous 3D CNT electrode that could resolve the problems with the Li metal anode, as illustrated in Figure 1a,b, in contrast to conventional cells utilizing commercial polypropylene (PP) separators (Figure 1c,d). In the past, most efforts have been devoted to the development of Li metal anodes with porous frameworks or separators. However, our series of simulations and experimental results have unveiled that such individual components are unlikely to address the detrimental problems, so it is necessary to introduce effects beyond what individual separators and anodes can offer. Our electronically insulating composite layer can delocalize Li^+^ flux passing through the PP separator. Our CNT was self-entangled during the synthesis process and formed into 3D porous structures having superb mechanical resiliency without any binders.^27,28^ The large contact area between the polymer layer and the CNT prevents delamination, which is crucial in eliminating the voids between the separator and anode. Conversely, conventional PP separators and anodes cannot preclude the formation of the voids in between, accelerating Li dendrite growth along with the localized Li^+^ transport through the PP separators (Figure 1d). The delocalized Li^+^ through our composite separator is readily inserted into the pores and then plated over the entire CNT surface, effectively inhibiting the clogging issue of the 3D host framework (Figure 1b). Through finite element analysis (FEA) numerical simulations, we theoretically predicted the different trends of Li^+^ concentration distribution across the separator in each case: from the commercial PP separator and 2D Li metal anode to the proposed SE composite separator and 3D CNT anode. Then, we thoroughly characterized each component, showing a high Li^+^ transference number of our composite separator and low Li nucleation potential of our 3D CNT anode as well as the roles of each component in the cell, unveiling that desirable outcomes can be obtained only when both the composite separator and the CNT anode were paired. Furthermore, full-cell tests with low- and high-voltage cathodes have demonstrated the practicality of our approach in boosting energy densities and cycling performances.

## Results and Discussion

Our composite separator and CNT anode were fabricated by coating a composite containing Li_6.4_La_3_Zr_1.4_Ta_0.6_O_12_ (LLZTO) and poly(vinylidene fluoride-*co*-hexafluoropropylene (PVDF-HFP) on a commercial polypropylene (PP) separator and then firmly attaching it to a CNT electrode, as described in the Supporting Information. We employed the PVDF-HFP polymer to bind LLZTO SE particles, because the pores of PVDF-HFP are copious and small enough to distribute Li^+^. The intrinsically low crystalline structure and strong electron-withdrawing functional group (−C–F) of PVDF-HFP^29−32^ lead to a high dielectric constant^33^ and a decent ionic conductivity at room temperature^34,35^ compared to other polymers, including poly(ethylene oxide),^36^ polyacrylonitrile,^37^ poly(methyl methacrylate),^38^ and polyimide.^39^ However, PVDF-HFP is unstable under a typical cycling voltage (>4 V) of Li-ion batteries.^40,41^ This shortcoming could be alleviated by blending ceramic fillers such as TiO_2_,^42^ SiO_2_,^43^ Al_2_O_3_,^44^ and BaTiO_3_^45^ and solid-state electrolyte particles including Li_1.5_Al_0.5_Ge_1.5_(PO_4_)_3_ (LAGP)^46^ and Li_7_La_3_Zr_2_O_12_ (LLZO)^47−53^ with PVDF-HFP. Here we have selected LLZTO to fabricate our flexible SE composites due to its high mechanical rigidity^54^ and Li^+^ attracting characteristic.^55,56^

**Figure 2:**
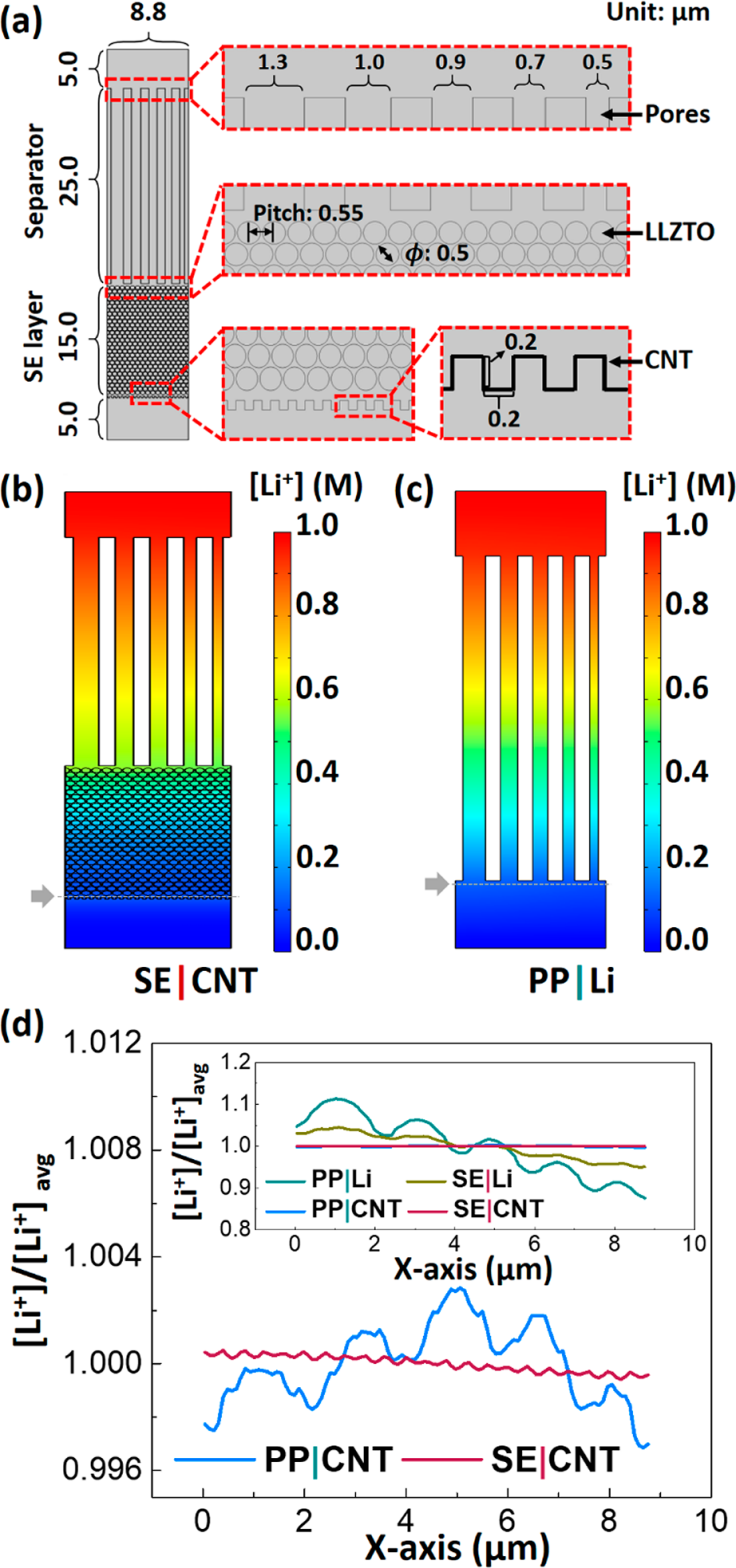
(a) FEA simulation geometry for the SE|CNT pair. Detailed information on other cases is summarized in Figure S1. Li^+^ concentration distribution through (b) SE|CNT and (c) PP|Li. Color gradients in each figure indicate the concentration of Li^+^ (M). Normalized Li^+^ concentrations for each case are calculated at the gray dotted line (gray arrow). (d) Normalized Li^+^ concentration for PP|CNT (blue line) and SE|CNT (red line). Results of other cases are represented in the inset figure (green line for PP|Li and yellow line for SE|Li, respectively). Li^+^ concentration calculation line at a distance of 1 μm beneath the separator for each pair was selected to normalize Li^+^ concentration.

FEA numerical simulations (COMSOL Multiphysics 6.0) were conducted to analyze the difference in the Li^+^ migration trend and concentration distribution for the case where the SE separator and CNT anode framework were applied compared to the case where the commercial PP separator and 2D Li metal anode were used. Detailed 2-dimensional geometry domain information for each separator|anode pair is summarized in Figure S1. The nonuniformly distributed vertical pores of the PP separator, spherical LLZTO particle arrays of the SE layer, and the large corrugated surface area of CNTs were applied to each simulation geometry domain, and the representative domain (SE|CNT) which exhibits all these characteristics is represented in Figure 2a. Information about governing equations and boundary conditions established for FEA simulations is summarized in the Supporting Information. For the PP|Li separator-anode pair case (Figure 2c), Li^+^ migrates from the cathode to the anode (vertically) through the liquid electrolyte filled in the nonuniform pores inside the PP separator. Due to the uneven pores and spacing between each pore, which does not contain the electrolyte, a normalized Li^+^ concentration, which was measured at 100 nm below the separator region, represented nonuniform distribution at the bottom of the PP separator. The normalized Li^+^ concentration distribution is shown in the inset of Figure 2d (green line). The result implies the nonuniform Li deposition on the 2D Li anode and thus promotes dendrite growth. On the contrary, in the case of SE|Li (Figure S2a), the normalized Li^+^ concentration shows more uniform and low fluctuation compared to the PP|Li case (yellow line in the inset of Figure 2d). This is attributed to the effect that Li^+^ migrated through the nonuniform pores of the PP separator to become homogenized while passing through the 3D conducting pathways of the LLZTO arrays in the SE composite membrane. It is well-known that the topological characteristics of garnet-type LLZTO, such as sufficient Li^+^ transport pathways through the grain boundaries^57^ and the crystal structures,^58,59^ give rise to this Li^+^ redistribution effect. Several previous studies have demonstrated that the grain boundaries with ion migration barriers and different Li^+^ conduction characteristics can affect Li^+^ transport heterogeneity of the solid-state electrolyte.^93,94^ However, the length scales associated with grain boundaries are much smaller than those of the pores of conventional separators. Thus, atomistic-scale ion transport heterogeneity was not considered in this work. Consequently, applying the SE separator results in less fluctuation of the normalized concentration. Normalized Li^+^ concentration with a small gradient implies more uniform Li^+^ flux near the anode surface and, thus, uniform Li deposition compared to the PP separator. Moreover, the corrugated-shaped CNT domain which has a 0.2 μm interval was designed for PP|CNT and SE|CNT pairs to simulate the large surface area (Figure S5) compared to the planar Li anode. Several previous studies have investigated that the use of a 3D anode framework with a large surface area guides uniform Li plating on its surface due to the local current density mitigation effect in the vicinity of the anode surface.^17−22^ By applying a large-surface-area CNT anode rather than a planar Li anode, PP|CNT (Figure S2b) showed significantly mitigated fluctuation of the normalized Li^+^ concentration caused by the nonuniform pores of the PP separator (blue line in Figure 2d). The standard deviation of Li^+^ concentration decreased to 1 order of magnitude by exploiting the CNT anode. According to this, we hypothesized that if the abundant 3D Li^+^ migration pathways of the SE separator and the Li^+^ concentration distribution homogenization effect of the 3D CNT anode are applied together, it can be anticipated to obtain a more improved Li^+^ redistribution effect. As a result, SE|CNT (Figure 2b) represented the most uniform normalized Li^+^ concentration (red line in Figure 2d) and the lowest standard deviation throughout all of the cases, which agrees well with the hypothesis. The standard deviation results of each separator|anode pair are summarized in Table S1. According to the simulation results, both stable cycling life and superior performance can be expected by utilizing the synergistic effect between the SE composite separator and the 3D CNT anode of the proposed Li metal battery in this work. FEA simulation results proposed in this work are only meaningful in predicting the ideal Li^+^ transfer mechanism for each separator-anode pair (e.g., PP|Li, PP|CNT, SE|Li, and SE|CNT). Specifically, to conduct a comparative analysis of the Li^+^ migration profile in the presence of the nanoporous structure of the SE separator, Nernst–Planck equations without considering the Faradaic reaction were applied as governing equations. The Nernst–Planck–Poisson equation may better simulate both Li^+^ migration propensity and Li plating kinetics at the anode surface, as it describes both Li^+^ transport and the electrochemical reaction. The results observed in actual practical cycling cases may differ from the simulation results. Nevertheless, by conducting these fundamental numerical simulations, we were able to theoretically predict and analyze the difference in the Li^+^ distribution effect for each pair and perform experimental validation.

**Figure 3:**
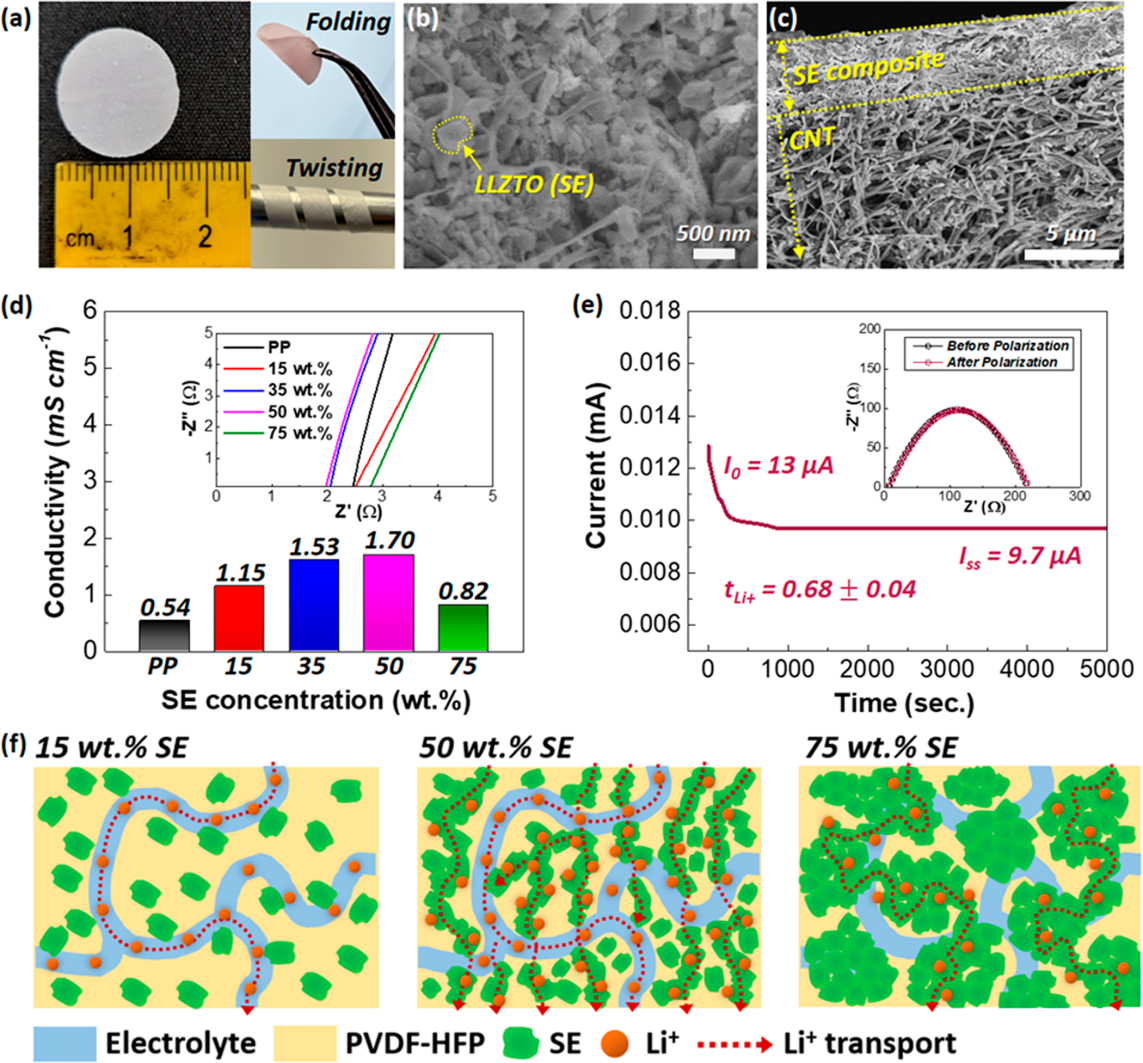
(a) Photographs of a 50 wt % SE composite film, showing unfolded, folded, and twisted morphologies to demonstrate mechanical flexibility. (b) Cross-sectional scanning electron microscope (SEM) image of the 50 wt % SE composite. (c) Cross-sectional SEM image of the SE composite integrated with the 3D CNT electrode, showing strong adhesion to avoid delamination. (d) Ionic conductivities (σ) of a PP separator and 15, 35, 50, and 75 wt % SE composites on the PP separator. σ was obtained from bulk resistance values (*R*_b_) which are the *x*-axis intercepts of EIS measurements as shown in the inset. (e) Chronoamperometry results of a Li|SE|Li cell with a 50 wt % SE composite on a PP separator under a static overpotential of 10 mV. The corresponding EIS plots before and after the polarization are shown in the inset. (f) Conceptual illustrations depicting Li^+^ conducting paths for the 15, 50, and 75 wt % SE composites. At a low SE concentration, Li^+^ predominantly migrates through tortuous pores, where the liquid electrolyte is filled. At a medium SE concentration, the percolated LLZTO particles can create more and shorter Li^+^ conducting paths in addition to conduction through the pores. When the SE concentration is too high, SE particles tend to aggregate, creating bottlenecks in the Li^+^ conducting paths. Some pores are blocked by the aggregated SE particles, deterring Li^+^ conduction through the pores.

Based on the simulation analysis for the synergistic effect of the SE|CNT pair under ideal conditions, the following experimental results were investigated to analyze the improved performance under practical conditions. Figure 3a displays fully dried composite films containing 50 wt % LLZTO and 50 wt % PVDF-HFP with a thickness of 15 μm, demonstrating mechanical flexibility. The cross-sectional image of the film (Figure 3b) revealed that the film is highly porous (∼80% porosity) and LLZTO particles of several hundred nanometers in size are uniformly distributed. When the composite film was integrated with the CNT anode, the large contact area between the polymer and CNT provides strong adhesion that prevents delamination, as shown in Figure 3c and Figure S6. The continuous 3D nanopores effectively disperse Li^+^ transport, unlike the conventional PP separators (Figure S7), where Li^+^ funnels through relatively large pores with low porosity (typically <50%). The top surface of our composite film (Figure S8) shows that LLZTO particles are embedded into the film and the pores are too small to be clearly seen on the top surface unlike the case for the PP separator. LLZTO allows for Li^+^ conduction through the percolated networks of LLZTO particles, furthering the distribution of Li^+^ in the composite layer, which is advantageous over other metal oxides (e.g., Al_2_O_3_, TiO_2_, SnO_2_, etc.).^60^ According to the X-ray diffraction (XRD) pattern (Figure S9a), all the peaks corresponding to LLZTO were maintained when LLZTO was made into composites with PVDF-HFP, suggesting that the properties of LLZTO are well retained.^61^ Fourier transform infrared radiation (FTIR) results (Figure S9b) indicate the addition of LLZTO into PVDF-HFP decreased the crystallinity of PVDF-HFP, which can improve the ionic conductivity of the composite.^62,63^

We first identified a suitable concentration of LLZTO in the composite by characterizing the ionic conductivities (σ) of the LLZTO composites on PP separators. The bulk resistance values in the electrochemical impedance spectroscopy (EIS) measurement (the intercept of the *x*-axis in the inset of Figure 3d) using a symmetrical cell configuration with stainless steel (SS) current collectors were used to find the conductivities with geometrical parameters (Table S2) using eq S1. The highest conductivity (1.7 mS cm^–1^) was measured at 50 wt % LLZTO, which is about 3 times higher than that of the PP separator (0.54 mS cm^–1^), as displayed in Figure 3d. Interestingly, the conductivity was raised with LLZTO contents up to 50 wt % and then dropped at 75 wt %. In our composites, lithium ions can conduct through both percolated solid-state electrolytes and pores where liquid electrolyte was filled up. An increase of SE wt % can augment percolated SE networks as long as the SE concentration is moderate. However, when the SE wt % is too high, the SE particles tend to aggregate due to the higher viscosity of the SE/polymer mixture solution. Then the aggregated SE reduces the contact area between SE particles and thereby suppresses the Li^+^ transport, as illustrated in Figure 3f. Therefore, 50 wt % LLZTO was selected for further experiments, although there may be room for further improvement in the conductivity with a variety of blending ratios of LLZTO to PVDF-HFP.

Another important parameter as a measure of Li^+^ transport through the SE composite layer is the Li^+^ transference number (*t*_Li^+^_) (see eq S2), which was obtained from the two *x*-intercepts of the semicircles of EIS (inset of Figure 3e) before and after chronoamperometry polarization (Figure 3e) using the symmetrical cell configuration with Li metal electrodes (see more details in the Supporting Information). The *t*_Li^+^_ value of the 50 wt % LLZTO composite on the PP separator was found to be 0.68, which is about 31% higher than the *t*_Li^+^_ value without the composite (only the PP separator), as shown in Figure S10. Sand’s time model^64^ generally described that the higher *t*_Li^+^_ indicates a longer Li dendrite growth time. Although there is difference between the interelectrode distance measured in this work and in Sand’s time model, the higher *t*_Li^+^_ of our SE composite layer can be realized as promoting uniform Li plating on the anode. The increase of *t*_Li^+^_ could be attributed to the following two aspects—Li^+^ transport paths through the SE networks which Li^+^ can selectively pass through and the negative ζ potential of LLZTO^55,56^ which can facilitate Li^+^ diffusion over anions such as PF_6_^–^ and DFOB^–^.^65−67^ In addition, the oxidation stability of the composite (50 wt % LLZTO) on the PP separator was measured to be high (∼4.9 V vs Li^+^/Li) enough to be compatible with the high-nickel cathodes such as Li[Ni_0.8_Mn_0.1_Co_0.1_]O_2_ (NMC811) according to linear sweep voltammetry (LSV) at a 5 mV/s scan rate (Figure S11).

**Figure 4:**
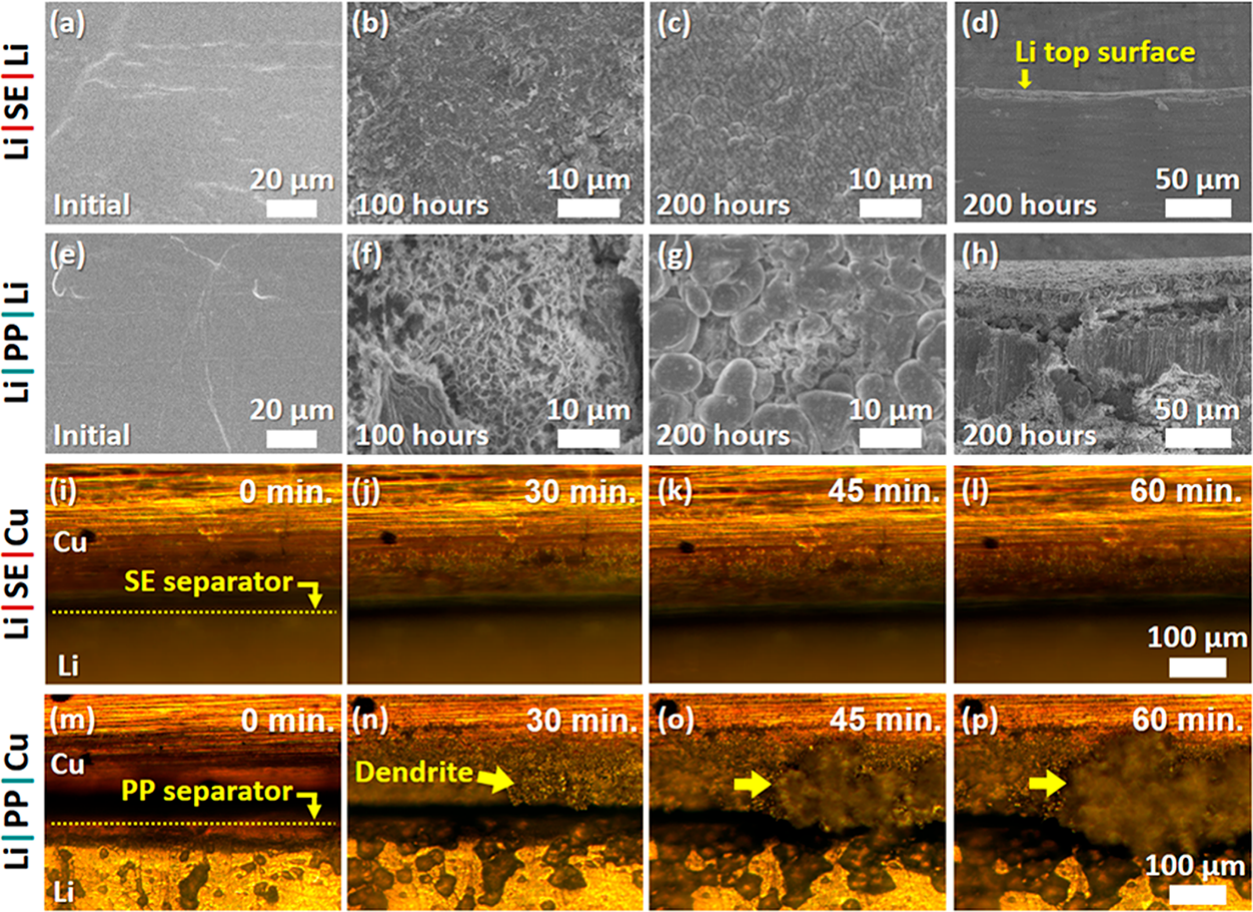
(a–d) SEM images of Li electrode surfaces of the Li|SE|Li cell (a) before cycling and (b) after 100 h (25th cycle) and (c) 200 h (50th cycle) cycling at a capacity of 2 mAh cm^–2^ and a current density of 1 mA cm^–2^. (d) Cross-section of the Li electrode in (c). The arrow indicates the surface shown in (c). (e–h) Li electrode surfaces of the Li|PP|Li cell (counterparts of (a–d)) (e) before cycling and after (f) 100 h and (g) 200 h cycling. (h) Cross-section of (g). The top surface clearly shows thick porous dendrite structures. (i–l) In-operando images of the Li|SE|Cu cell (i) before Li plating and after Li plating on the Cu surface for (j) 30, (k) 45, and (l) 60 min at a current density of 4 mA cm^–2^ and a capacity of 4 mAh cm^–2^, displaying uniform Li plating with the SE composite. (m–p) In-operando images of the Li|PP|Cu cell (counterparts of (i–l)) (m) before Li plating and after Li plating on the Cu surface for (n) 30, (o) 45, and (p) 60 min, revealing nonuniform Li plating with growing dendrites on the Cu surface and the extremely rough Li metal surface.

The actual Li plating/stripping behaviors were analyzed when our SE composite separator was used in symmetrical cells with Li metal foils as electrodes. All the following tests were carried out with 50 wt % LLZTO for our SE composite unless otherwise noted. The surface morphologies of the Li metal anodes before and after cycling (100 and 200 h) at a current density of 1 mA cm^–2^ and a capacity of 2 mAh cm^–2^ were inspected when our SE composite separator (Figure 4a–d) was employed (Li|SE|Li cell) in comparison to a conventional PP separator (Li|PP|Li cell) (Figure 4e–h). Cycling over 100 h did not noticeably alter the Li metal surface of Li|SE|Li (Figure 4b), in contrast to the nonuniformly distributed branch-like dendrites from Li|PP|Li (Figure 4f). After 200 h of cycling, the dendrites on Li|PP|Li grew noticeably (Figure 4g), whereas Li|SE|Li maintained a uniform and dense surface morphology (Figure 4c). These features were manifested in the cross-sectional views showing a dense and thin layer from Li|SE|Li (Figure 4d) and a porous and thick layer from Li|PP|Li (Figure 4h) on the Li foils. The uniform Li plating/stripping is ascribed to delocalized Li^+^ flux through the SE layer, but in Li|PP|Li, the Li^+^ flux through the pores of the conventional PP separator is concentrated, accelerating dendrite nucleation and growth.

The Li homogenization effect with the SE composite was further validated using operando studies by plating Li on a Cu current collector at a high current density of 4 mA cm^–2^ and a capacity of 4 mAh cm^–2^, as displayed in Figure 4i–l for Li|SE|Cu and Figure 4m–p for Li|PP|Cu asymmetrical cells (see the Supporting Information for the operando cell test). As for the Li|SE|Cu cell, even under a high current density (4 mA cm^–2^), the morphology variations of plated Li on the Cu current collector were hardly noticed throughout the entire plating (60 min), as shown in Movie 1 in the Supporting Information along with voltage profiles corresponding to all the operando images in Figure S12. In contrast, the Li|PP|Cu cell localized Li plating (Figure 4n), and then dendrites (see the spot indicated by the arrow) rapidly grew as a result of preferential Li deposition on the dendrites (Movie 2 in the Supporting Information). The dendrite after 4 mAh cm^–2^ Li plating was too large to be within the depth of focus. Another image in focus is available in Figure S13. Interestingly, the Li metal surface of Li|PP|Cu became very rough, evidently demonstrating that Li^+^ flux through the PP separator was nonuniform and detrimental during Li plating and stripping.

**Figure 5:**
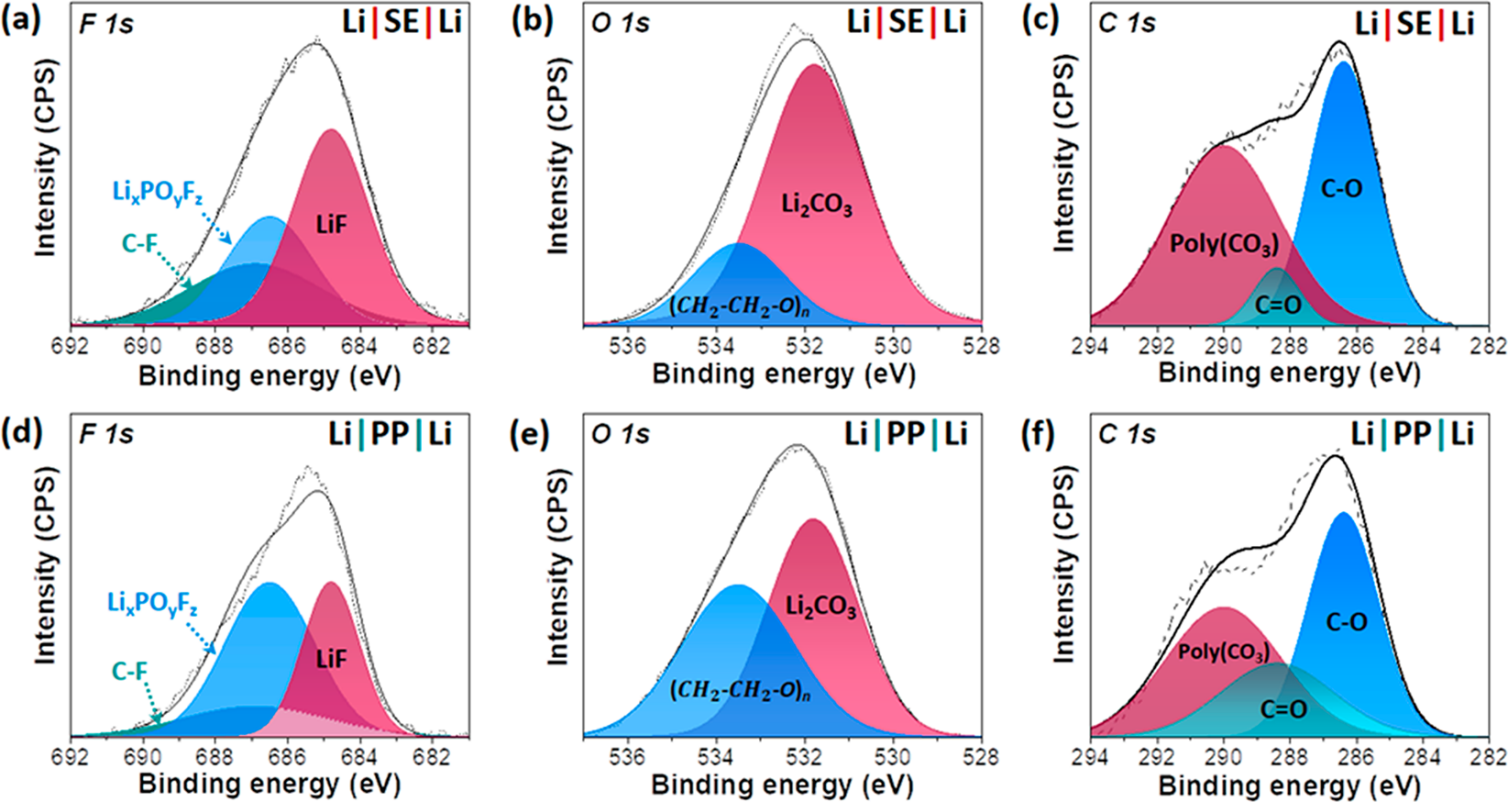
XPS results of the Li metal surfaces of (a–c) the Li|SE|Li cell and (d–f) the Li|PP|Li cell corresponding to F 1s, O 1s, and C 1s after 100 h cycling at a current density of 1 mA cm^–2^ and a capacity of 2 mAh cm^–2^.

The compositional variations as a result of delocalizing Li^+^ flux were comparatively investigated by X-ray photoemission spectroscopy (XPS) of Li metal surfaces after 100 h operation of Li|SE|Li (Figure 5a–c) and Li|PP|Li (Figure 5d–f) symmetrical cells. During the cycling, solid electrolyte interphase (SEI) layers were formed and their F 1s, O 1s, and C 1s peaks were evaluated. We noticed that the areal ratio corresponding to the Li–F peak (684.8 eV)^68,69^ in F 1s from the Li|SE|Li cell was larger (∼48%) than that of the Li|PP|Li cell (∼33%). The LiF-rich SEI layer was found to promote uniform Li plating and suppress the corrosion of Li because it has outstanding physiochemical stability due to the high Young’s modulus (64.9 GPa).^70,71^ Another stable component, Li_2_CO_3_ (531.8 eV)^71^ from O 1s in the SEI layer of Li|SE|Li (∼79%), was more abundant than that of Li|PP|Li (∼54%). It is known that the lithium difluoro(oxalato)borate (LiDFOB) additive in the carbonate-ester electrolyte used in this work boosts stable inorganic components (e.g., LiF, Li_2_CO_3_) in the SEI layer.^72,73^ In addition, the areal ratio of polycarbonate (poly(CO_3_) in C 1s at 290.0 eV),^74,75^ which suppresses the swelling of Li during plating/stripping,^74^ was measured to be higher (∼49%) for Li|SE|Li compared with Li|PP|Li (∼39%), suggesting that the delocalization of Li^+^ flux and high Li^+^ transference number are crucial in forming stable SEI layers.

In the same vein, the areal ratio corresponding to Li_*x*_PO_*y*_F_*z*_ in F 1s (686.5 eV),^76^ which is attributed to the undesirable decomposition of LiPF_6_ salt,^77−79^ was found to be lower (∼28%) in Li|SE|Li than that of Li|PP|Li (∼51%). The result is consistent with the areal ratio of C–F bonding in F 1s (686.9 eV),^80^ which is attributed to the decomposition of fluoroethylene carbonate, being found to be higher (∼24%) in Li|SE|Li compared to the Li|PP|Li (∼16%). Another unwanted byproduct,  (533.5 eV),^75^ from the decomposition of carbonate electrolyte^75^ lessened with the SE layer (∼22%) compared to ∼46% with only the PP separator. Moreover, the total of organic components in the Li|SE|Li cell is ∼51% (45% for C–O at 286.4 eV and 6% for C=O at 288.4 eV),^81^ which is ∼10% lower than that of the Li|PP|Li cell (∼61%). The aforementioned XPS analyses indicate that the SE layer made it possible to create more stable SEI layers, which impeded dendrite growth owing to the delocalized Li^+^ conduction.

Although the uniform Li^+^ flux can delay the dendrite growth on the 2D Li metal anode, the limited surface area of 2D Li metal intrinsically makes it difficult to irradiate the problems during the cycling process. Here we further studied the effects of having a 3D host framework as a Li metal anode, which can induce even more uniform Li plating and further suppress dendrite growth. In particular, the nanostructured 3D CNT framework offers large specific surface areas that lower the local current density, regulating the substantial volume change of Li metal in the anode during Li plating/stripping. Here, we utilized previously reported functionalized unzipped CNT structures which were fabricated by opening C–C bonds with the formation of manganate ester due to the lower bonding energy between the MnO_4_^–^ anion and carbon. The partially unzipped trench CNTs have hybridized lithiophobic/-philic surfaces by attaching carboxyl (−COOH) functional groups to the cleaved CNT surfaces,^82^ which offer an extremely large capacity (∼16 mAh cm^–2^) at a high current density (8 mA cm^–2^) without dendrite formation.^82−84^ As demonstrated in Figure 3c, the large interfacial surface area of the 3D CNT anode with an additionally unzipped trench structure induced strong adhesion between the SE composite and the 3D CNT anode.

**Figure 6:**
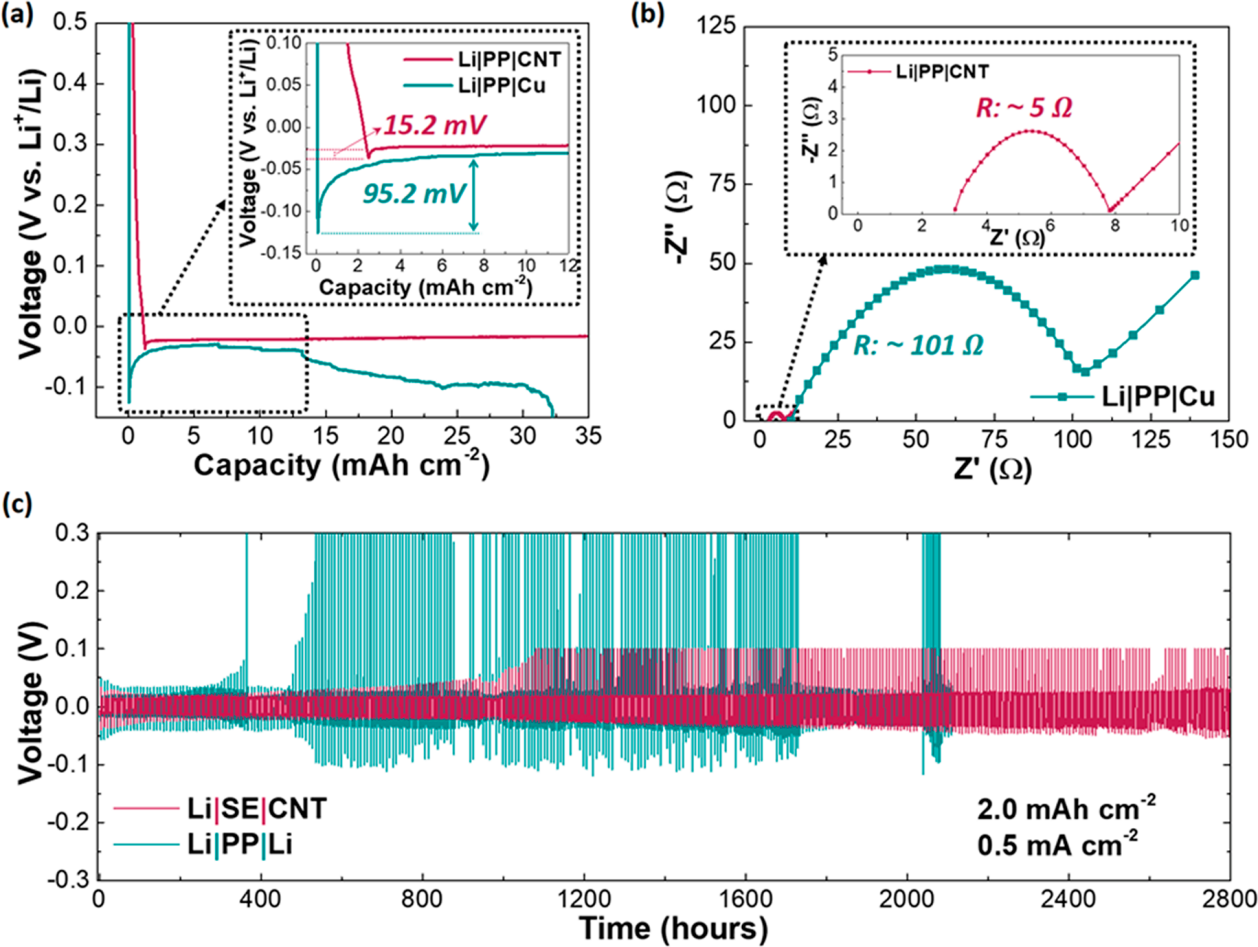
(a) Voltage profiles of the Li|PP|CNT and Li|PP|Cu cells during Li plating at a current density of 1 mA cm^–2^ until the capacities reached 40 mAh cm^–2^. The inset shows the nucleation overpotentials. (b) Nyquist plots of the Li|PP|CNT cell (the inset shows an enlarged plot) and the Li|PP|Cu cell after the 5th plating/stripping cycle (2 mAh cm^–2^ and 1 mA cm^–2^). (c) Galvanostatic Li plating/stripping voltage profiles of the Li|SE|CNT and Li|PP|Li cells (2 mAh cm^–2^ and 0.5 mA cm^–2^).

The effects of employing CNTs were evaluated by assessing the galvanostatic voltage profiles during Li plating (Figure 6a) and EIS measurement results at 5 mV after Li stripping from the CNT electrode (Figure 6b) for Li|PP|CNT and Li|PP|Cu asymmetrical cells. As witnessed in the operando study (Figure 4m–p), the Li|PP|Cu cell showed high nucleation overpotential (η_nu_ ≈ 95 mV), which can be calculated as the difference between the tip overpotential (η_tip_ ≈ 125 mV) and the converged mass-transfer-controlled overpotential (η_mtc_ ≈ 30 mV).^85^ When the amount of Li deposition exceeded 13 mAh cm^–2^, a gradual increase in η_mtc_ occurred, and after 30 mAh cm^–2^ of Li deposition, a drastic increase in the overpotential was observed. The large overpotential and unstable voltage profile of the Li|PP|Cu cell denote the nonuniformly deposited Li on the Cu surface. On the contrary, it is clearly seen that η_nu_ (∼15 mV) from Li|PP|CNT was much smaller than that from Li|PP|Cu. The voltage dropped as lithiation progressed and then became negative, indicating lithium metal was plated into the pores of the 3D CNT framework beyond lithiation. Despite the large amount of Li insertion (∼35 mAh cm^–2^) into the CNTs, η_mtc_ was maintained at ∼21 mV, showing stable η_nu_ values. The stable voltage profile of the Li|PP|CNT cell can be attributed to the uniformly deposited Li because the carboxyl group attached to the partially unzipped CNT surface can attract Li^+^, unlike pristine graphitic carbon.^86,87^ The reduced current density with the CNT electrode was confirmed by the EIS results of Li|PP|CNT in comparison to those of Li|PP|Cu asymmetrical cells (Figure 6b). The diameter of the semicircle denotes impedance associated with charge transfer at the surface of the electrode. The impedance of Li|PP|CNT was measured to be ∼5 Ω, which is only ∼5% compared to that of Li|PP|Cu (∼101 Ω).

As the CNT in the Li|PP|CNT cell facilitated uniform Li plating on the CNT anode, we carried out long-term testing of our SE composite separator (Li|SE|CNT asymmetrical cell) along with a Li|PP|Li cell for comparison. The galvanostatic cycling stability tests were conducted with a capacity of 2 mAh cm^–2^ at 0.5 mA cm^–2^ (Figure 6c and Figures S14 and S15), 1 mA cm^–2^ (Figure S16), and 2 mA cm^–2^ (Figure S17). The overpotentials during plating/stripping of Li into/from CNT at 0.5 mA cm^–2^ were ∼13 mV over ∼800 h (∼100 cycles), and then the stripping overpotential was slightly enlarged to 0.1 V and maintained over 2800 h (red lines in Figure 6c). The stripped-to-plated lithium metal ratio for Li|SE|CNT was maintained at ∼98%, which supports the stable plating/stripping behavior of the SE|CNT pair compared to PP|Li. In contrast, the voltage profile of the Li|PP|Li cell was unstable, showing large overpotentials at only ∼500 h of operation (∼60 cycles). A higher current density of 1 mA cm^–2^ did not noticeably alter Li plating/stripping behaviors of Li|SE|CNT, while the cycling lifetime of Li|PP|Li was considerably shortened (Figure S16). The superior cycling stability of Li|SE|CNT compared to Li|PP|Li is indicative of the synergistic effect from the SE layer and the 3D CNT host framework. The delocalized Li^+^ flux through the SE layer facilitates homogeneous Li plating, and the large surface area of CNT lowers the areal current density and thereby promotes uniform Li plating/stripping, reducing charge transfer impedance and dendrite formation.

**Figure 7:**
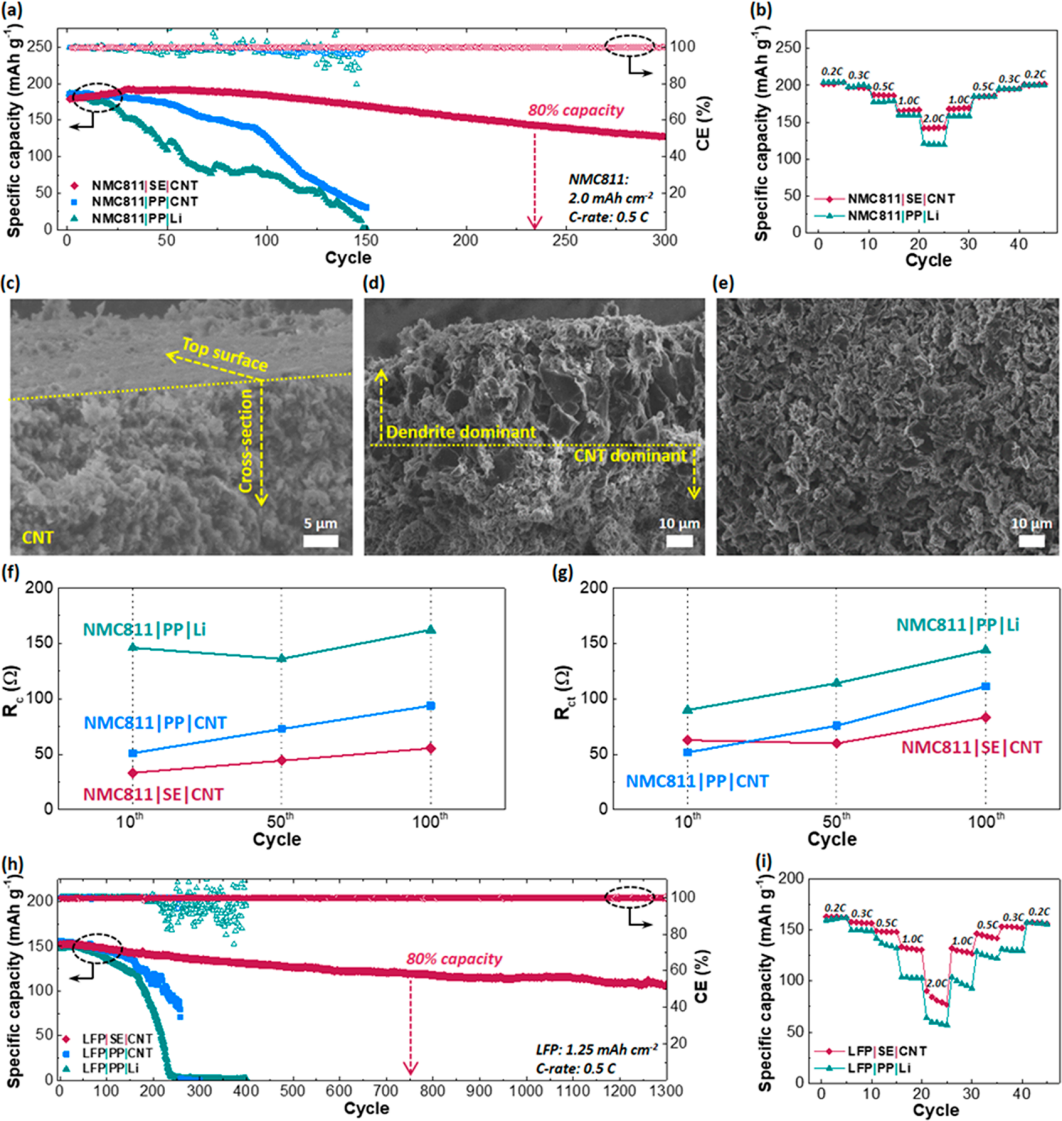
(a) Specific discharge capacities (filled symbols) and Coulombic efficiencies (CE) (hollow symbols) for NMC811|SE|CNT (red diamonds), NMC811|PP|CNT (blue squares), and NMC811|PP|Li (green triangles) full cells at 0.5 C with a voltage window of 2.8–4.3 V. The areal capacity of the NMC811 cathode is 2 mAh cm^–2^. The negative to positive electrode capacity ratios (N/P ratios) are 5.0, 5.0, and 6.7 for NMC811|SE|CNT, NMC811|PP|CNT, and NMC811|PP|Li, respectively. (b) Rate capabilities of NMC811|SE|CNT and NMC811|PP|Li at 0.2 0.3, 0.5, 1.0, and 2.0 C. (c) Cross-sectional view of the 3D CNT anode of the NMC811|SE|CNT full cell at the fully discharged state after the 100th cycle, showing both the cross-section (bottom part) and surface facing the separator (top part). (d) Cross-sectional view of the 3D CNT anode of the NMC811|PP|CNT full cell at the fully discharged state after the 100th cycle, showing a Li-dendrite-rich portion (top) and a CNT-rich portion (bottom). (e) CNT surface facing the separator (same sample as in (d)). (f) Interfacial contact impedance (*R*_c_) and (g) charge transfer impedance (*R*_ct_) of NMC811|SE|CNT, NMC811|PP|CNT, and NMC811|PP|Li at the fully discharged state of the 10th, 50th, and 100th cycle. The cycling conditions are the same as those in (a). (h) Specific discharge capacities (filled symbols) and CEs (hollow symbols) for the LFP|SE|CNT (red), LFP|PP|CNT (blue), and LFP|PP|Li (green) full cells at 0.5 C with a voltage window of 2.5–4.1 V. The areal capacity of the LFP cathode layer is 1.25 mAh cm^–2^, and the N/P ratios are 6.4, 6.4, and 10.7 for LFP|SE|CNT, LFP|PP|CNT, and LFP|PP|Li, respectively. (i) Rate capabilities of LFP|SE|CNT and LFP|PP|Li at 0.2, 0.3, 0.5, 1.0, and 2.0 C.

To validate the practical applicability of the SE layer and 3D Li-deposited CNT anode, full-cell tests with the most representative metal oxide based cathodes, NMC811 and LiFePO_4_ (LFP) in Li-ion batteries, were carried out. All tests were conducted after initial SEI-formation cycles (3 cycles at 0.1 C where 1 C = 200 mA g^–1^ for NMC811 and 1 C = 170 mA g^–1^ for LFP). Figure 7a displays cycling performances at 1 mA cm^–2^ (0.5 C) when NMC811 (active material loading of ∼12.1 mg cm^–2^) was coupled with a SE/CNT (separator/anode) pair, a PP/CNT pair, and a PP/Li pair with a charge/discharge voltage window of 2.8–4.3 V. Forthe NMC811|SE|CNT cell (red diamonds), the initial discharge capacity was ∼179 mAh g^–1^ and gradually increased to 192 mAh g^–1^ at the 40th cycle, presumably due to the initial activation of the SE layer. After this cycle, the capacity fading was only ∼0.19 mAh g^–1^ (∼0.1%) per cycle with an excellent average CE (∼99.8%), and 80% capacity retention with respect to the initial capacity was marked at the 235th cycle (also see voltage profiles in Figure S18a). When the PP and Li metal foil (NMC811|PP|Li, green triangles) were used, the capacity rapidly decayed, showing 80% capacity retention (149 mAh g^–1^) with respect to the initial discharge capacity (186 mAh g^–1^) at only the 35th cycle. Large increases in the cell overpotential (Figure S18c) and CE fluctuation were observed in the following cycles, and eventually, the capacity became almost zero at the 150th cycle. The limited surface area on the 2D Li metal anode and localized Li^+^ flux would be responsible for the poor cycling performances, as corroborated by nonuniform dendrites and dead lithium on the anode as in Figure 4g.

When 2D Li metal was replaced by CNT without the SE layer (NMC811|PP|CNT, blue squares), the cycling number at 80% capacity retention was prolonged from 35 to 80, but rapid capacity fading in the subsequent cycles was unavoidable (also see voltage profiles in Figure S18b). The extended cycling stability would benefit from the reduced current density on the CNT surface. Nevertheless, the localized Li^+^ flux through the PP separator gradually formed Li dendrites during repeated Li plating/stripping. Figure 7b shows a rate capability comparison between NMC811|SE|CNT and NMC811|PP|Li. The average capacity data for each current density of the two cells are summarized in Table S3. The average discharge capacity between the two cases shows a small difference under the low-C-rate conditions (0.2, 0.3, and 0.5 C). However, under the high-C-rate conditions (1.0 and 2.0 C), NMC811|SE|CNT demonstrated 6.5% (for 1.0 C) and 18.3% (for 2.0 C) higher average capacity results than NMC811|PP|Li. The higher rate capability of NMC811|SE|CNT cell can be attributed to the influence of the high *t*_Li^+^_ value of the SE composite separator and the reduced local current density effect from the CNT anode.

Additionally, the synergistic effects of SE composites and CNT anodes were further verified under practical conditions^83,84^ seeking high energy densities with high-loading NMC811 cathodes (4 mAh cm^–2^, active material loading of ∼21 mg cm^–2^) with a low N/P ratio (2.5). Three different types of separator/anode pairs (NMC811|SE|CNT, NMC811|PP|CNT, and NMC811|PP|Li) were comparatively tested. Their specific capacities over 200 cycles are displayed in Figure S19. The NMC811|SE|CNT full cell showed an outstanding maximum capacity (204 mAh g^–1^) at the 40th cycle despite a high current density (1.2 mAh cm^–2^) as well as ∼81% capacity retention with respect to the initial capacity (193 mAh g^–1^) at the 200th cycle. Contrariwise, the NMC811|PP|CNT and NMC811|PP|Li cells had 80% capacity retention at only the 113th and 117th cycles, respectively. Compared to the NMC811|SE|CNT cell, the NMC811|PP|CNT and the NMC811|PP|Li cells both showed rapid capacity fading and unstable cycling performances, which are consistent with the trend shown in Figure 7a (cathode capacity of 2 mAh cm^–2^).

The CNT anodes of NMC811|SE|CNT (Figure 7c) and NMC811|PP|CNT (Figure 7d for a cross-section and Figure 7e for a top surface facing the separator) at the 100th fully discharged state were inspected by SEM. The top portion in Figure 7c is the surface of the anode facing the separator, showing a flat surface without noticeable dendrites over the surface. The bottom portion is a cross-section exhibiting hairy CNT features and pores. The SE layer can distribute Li^+^ flux, so Li was inserted into the pores without considerably plating Li metal on the top surface. As revealed in Figure 3c, the hairy CNTs were well integrated into the SE layer, eliminating spaces for dendrite growth between the separator and the CNT anode. On the contrary, the cross-section of the anode in Figure 7d revealed that porous dendrites were formed over the CNT electrode, clearly depicting the role of the SE layer. The localized Li^+^ flux through the PP separator eventually clogged the pores of the anode surface and ended up with porous dendrites after repeated Li plating/stripping. The concentrated Li^+^ through the PP separator can be readily plated in the gap between the separator and anode, displaying a porous and uneven anode surface, as shown in Figure 7e. After clogging the pores of the CNT framework, the anode acts like a typical 2D Li surface without utilizing the inner pores. These experimental outcomes highlight the synergistic integration of our SE separator and CNT electrode, which can augment the cycling performance of the Li batteries.

The comparative analysis of the EIS measurement results (Figure 7f,g, Figure S20, and Tables S5–S7) at the fully discharged 10th, 50th, and 100th cycles for NMC811|SE|CNT, NMC811|PP|CNT, and NMC811|PP|Li further unveiled the effects of the SE layer (vs PP) and CNT framework (vs 2D Li metal). The equivalent circuit for EIS fitting (Figure S20a) consists of bulk impedance (*R*_b_), the interfacial contact impedance at the SEI layer (*R*_c_), and the charge transfer impedance at the electrolyte/electrode interface (*R*_ct_). The impedance values of NMC811|SE|CNT were kept low compared to those of the other cells, validating the synergistic effect from the SE layer and CNT framework. *R*_c_ with CNT was much lower than that with 2D Li metal even without the SE layer (Figure 7f), which elucidates the effectiveness of Li plating/stripping over the large surface areas. Moreover, *R*_ct_ of NMC811|PP|Li was considerably raised at the 100th cycle (Figure 7g), although all of the *R*_ct_ values at the 10th cycle were relatively close to each other. This would suggest the formation of dead Li on the porous Li metal anode as it impedes charge transfer at the electrolyte/electrode interface.

To identify the long-term stability of our composite and CNT pair, another set of full cells with LFP cathodes (active material loading ∼7.3 mg cm^–2^) was examined because LFP is known to be more long-lasting than NMC811. This experimental set does not only avoid cathode-limited performances but also verifies the compatibility of our separator/anode pair with another popular cathode. Figure 7h shows the cycling performances at 0.5 C (0.63 mA cm^–2^) with a potential window of 2.5–4.1 V. LFP|SE|CNT had high capacity retention with respect to the initial capacity of 152 mAh g^–1^, exhibiting ∼80% retention at the 750th cycle and a high average CE of ∼99.7% as well as ∼70% retention at the 1300th cycle (see voltage profiles in Figure S21). In contrast, LFP|PP|Li displayed a drastic capacity drop accompanied by large fluctuation in CE with ∼80% capacity retention (with respect to the initial capacity of 148 mAh g^–1^) at only the 160th cycle. When CNT replaced 2D Li metal (LFP|PP|CNT cell), the capacity retention was more or less improved (∼80% at the ∼200th cycle with ∼99.9% CE), but this cell suddenly failed to cycle at the 260th cycle. It is worth noting that the PP|Li and PP|CNT pairs showed substantially enlarged overpotentials as cycled according to the charge/discharge voltage profiles (Figures S18b,c and S21b,c) of the NMC and LFP full cells. At the cycles near the failure, the charge/discharge curves (Figures S22b,c and S23b,c) eventually became unsteady. The enlarged overpotentials for both charge and discharge imply that irreversible products such as dead Li covered the surface of the anode, inhibiting the transport of Li^+^ between the cathode and anode. Conversely, the SE|CNT pairs yielded stable voltage profiles near the PP|CNT and PP|Li failure (Figures S22a and S23a). Our observation suggests that the pores in the porous scaffolds are easily clogged over repeated nonuniform Li plating/stripping, which makes such porous structures eventually similar to 2D Li metal without the SE layer. The rate capability test results of the LFP full cells (Figure 7i) with the SE|CNT pair are also superior to those of the PP|Li counterpart particularly under high-C-rate conditions. The average capacity data corresponding to the C rates (current density) are arranged in Table S4. In addition to the NMC811 full cell test, the LFP test results elucidated the versatility of the SE composite separator and CNT anode with outstanding cycling performances.

According to the Li dendrite growth model derived from the Volmer–Weber theory,^95^ 3D substrates with large surface areas induce smooth Li plating compared to plain surfaces. Another study has investigated the correlation between the surface energy stabilization effect by exploiting a linear stability computational simulation.^96^ According to previous studies, the surface energy of 3D porous anodes is crucial in Li metal plating/stripping.^83,97^ For example, our previous studies elucidated that pristine graphitic surfaces on CNT are lithiophobic, which is disadvantageous in Li plating. On the other hand, lithiophilic carboxyl/hydroxyl functional groups on the graphitic surface of CNT can readily attract and diffuse Li^+^ into the porous structure of the 3D CNT. However, the Li^+^ affinity of the functional groups provoked pore clogging at the inlet (between the anode and separator) and eventually dendrite growth along with an inferior charge transfer compared to pristine graphitic surfaces. To mitigate this problem, mechanochemical treatments partially creating lithiophilic functional groups along the unzipped graphitic carbon surfaces were exploited. The favorable charge transfer of the preserved graphitic layer and presence of the lithiophilic functional groups allows for the intercalation and uniform deposition of Li on the CNT. Here, we have utilized the lithiophilic/lithiophobic hybridized CNTs as our 3D framework to have a uniform Li metal plating, which was validated by our experimental results such as low impedance results of Li|PP|CNT compared to Li|PP|Cu (Figure 6b), a smooth CNT anode surface without dendrites after the 100th cycle (Figure 7c), and EIS results of SE|CNT paired with the NMC811 cathode (Figure 7f,g).

**Figure 8:**
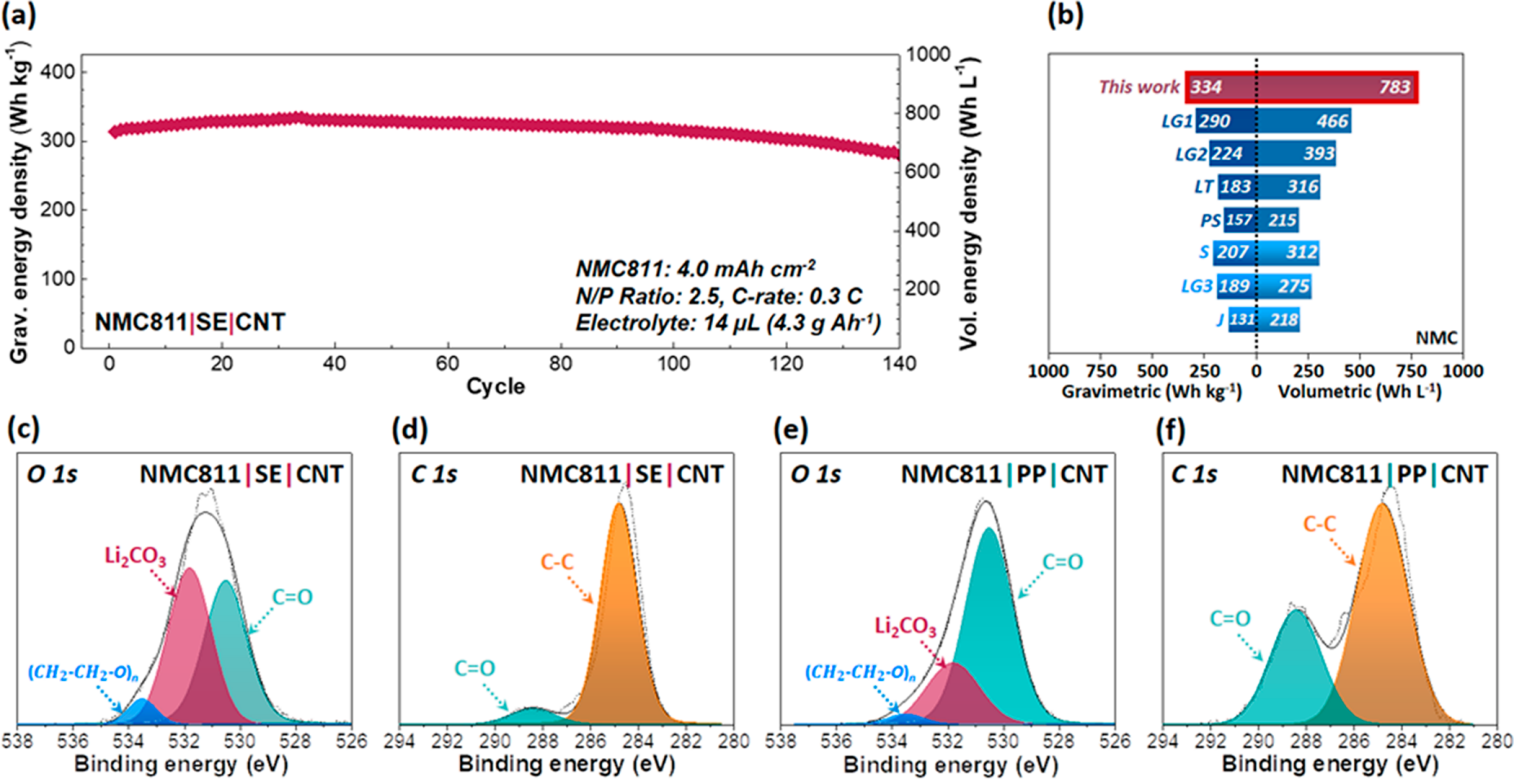
(a) Gravimetric (left *y*-axis) and volumetric (right *y*-axis) energy densities of the NMC811|SE|CNT full cell with a lean electrolyte condition (14 μL electrolyte, ∼4.3 g Ah^–1^) and a cathode with the areal capacity of 4 mAh cm^–2^ at 0.3 C (voltage window of 2.8–4.3 V). (b) Gravimetric (left) and volumetric (right) energy densities of NMC811|SE|CNT and LFP|SE|CNT (this work) in comparison to those of commercial cells (see more details in the Supporting Information and Table S8). The energy density values are based on electrodes, current collectors, electrolyte, and separator except for outer housing/casing (unavailable data). The symbols in (b) indicate the commercial cell suppliers. (S, Samsung SDI; LG, LG Chem.; J, Li Energy Japan; LT, Li-Tec; PS, Panasonic/Sanyo). XPS spectra corresponding to (c) O 1s and (d) C 1s peaks from NMC811|SE|CNT and (e) O 1s and (f) C 1s peaks from NMC811|PP|CNT at the fully discharged state of the 100th cycle under 2 mAh cm^–2^ and 1 mA cm^–2^ cycling conditions.

The gravimetric and volumetric energy densities of the NMC811|SE|CNT full cell (Figure 8a) were obtained, and the maximum gravimetric energy density was compared to those of popular commercial cells based on NMC cathodes (Figure 8b). The energy density values were attained by considering the mass and volume of the cathode, anode, separator, electrolyte, and current collectors (typical thicknesses of Al and Cu) except the outer housing/case (see the Supporting Information for more details about energy density calculation). The energy density values of the commercial cells (Table S8) were calculated based on the available performance data (e.g., specific energy (Wh kg^–1^), capacity (Ah), and working voltage (V))^88−90^ and the weight fraction of the cell components.^91^ Our NMC811|SE|CNT cell exhibited 334 Wh kg^–1^ and 783 Wh L^–1^, and the capacity retention was 89% at the 140th cycle even under practical conditions (N/P ratio 2.5, 4.3 g Ah^–1^ of the electrolyte). The gravimetric and volumetric energy densities are superior to those of commercial cells whose cathodes are made of NMC or NMC with lithium manganese oxide (LMO) along with graphite anodes. Furthermore, the cell-level energy densities of our NMC811|SE|CNT full cell are within the target range when the Li metal anode is coupled with NMC cathodes.^92^ This implies that the synergistic effect from our composite separator and CNT host anode would be a potential solution for utilizing Li metal as an anode. One of the previous studies revealed that the microstructural heterogeneity of the cathode, which is induced by the compositional ratio and particle sizes of the active material, has a significant effect on spatially uneven current and Li^+^ transport during charging/discharging.^98^ Therefore, it is anticipated that further Li^+^ flux delocalization could be obtained when the cathode design is configured along with our proposed separator/anode pair. The XPS analyses (Figure 8c–f) of the anode of NMC811|SE|CNT in comparison to those of NMC811|PP|CNT suggest that the superior performances of our full cell could be attributed mainly to the delocalization of Li^+^ plating/stripping on the anode side according to O 1s and C 1s spectra at the 100th fully discharged state of the full cells. We found that a stable inorganic component, Li_2_CO_3_ at 531.8 eV (O 1s), was higher in NMC811|SE|CNT (areal ratio: 47.9%) compared to NMC811|PP|CNT (24.5%). Furthermore, an unstable organic component, C=O at 288.4 eV (C 1s), was lower in the SE cell (7.6%) than in PP (33.9%).

## Conclusions

This work presents a feasible route of utilizing Li metal as an anode, which is considered to be the most practical option without altering the cathode and electrolyte of the current commercial Li-ion batteries. A Li metal anode could offer the highest energy density for conventional Li-ion batteries as long as the detrimental side effects such as severe capacity fading and Li dendrite formation can be mitigated. Here we identified the main failure mechanism when Li metal was substituted for the graphite anode of typical Li-ion batteries. When Li^+^ flux passes through the limited pores of the conventional PP separator, Li^+^ is inhomogeneously distributed, facilitating preferential Li plating and thereby promoting dendrite growth. Our composite separator consisting of SE (LLZTO) particles and polymer (PVDF-HFP) can provide numerous Li^+^ passages through the percolated SE and pore networks and thereby deliver delocalized Li^+^ to the anode. FEA simulation results for PP|Li and SE|Li theoretically confirmed that a more uniform Li^+^ concentration distribution was formed when the SE composite separator was utilized compared with the conventional PP separator. In addition, our composite separator has a higher conductivity and Li^+^ transference number in comparison to the PP separator. The operando and microscopy results revealed that the Li|SE|Li cell shows more uniformly distributed and compact Li metal surfaces after cycling compared with those of the Li|PP|Li cell. The XPS results also displayed that a robust and stable inorganic-rich SEI layer was formed on the Li anode by adopting SE and CNT according to the areal ratios of the Li–F and the Li_2_CO_3_ peaks.

A Li metal anode is also fatal when the SEI layer frequently breaks as a result of large volume changes over the limited 2D Li metal surface, because this is accompanied by a porous Li layer and Li consumption. Our 3D CNT framework anode furnished large surface areas, significantly reducing the current density and the volume change during Li plating/stripping. Through the FEA simulation, the SE|CNT case showed the lowest standard deviation of Li^+^ near the anode through all separator|anode pairs, which implies a superior Li^+^ homogenization effect and, thus, uniform Li plating behavior. The galvanostatic cell overpotential profiles and EIS data showed that the SE|CNT pair has a smaller nucleation overpotential (∼15 mV) and contact impedance (∼5 Ω) compared to those of the PP|Li pair, suggesting more uniform Li plating on the 3D CNT. Besides, the Li|SE|CNT asymmetrical cell retained low cell overpotentials and a high average CE over ∼2800 h of cycling. It should be pointed out that the stable operation of Li metal was observed only when both the SE and CNT were paired, suggesting that the synergistic effect is essential.

Finally, full-cell tests with the commercially most popular cathodes were carried out by coupling our SE|CNT pair. The NMC811|SE|CNT full cell showed ∼80% capacity retention with an average CE of ∼99.8% until the 235th cycle, whereas the NMC811|PP|Li cell experienced rapid capacity fading (∼80% capacity retention at the 35th cycle) with an unstable CE. Furthermore, the LFP|SE|CNT cell showed superior cycling performances (∼80% capacity retention at 750th cycle) with an average CE of ∼99.8%. According to EIS analysis, NMC811|SE|CNT possessed both low interfacial contact impedance (*R*_c_) and charge transfer impedance (*R*_ct_) compared to NMC811|PP|Li. The SEM images disclosed smooth anode surfaces for NMC811|SE|CNT in contrast to the dendrite-dominant clogged anode surface of NMC811|PP|CNT. Ultimately, the energy densities (334 Wh kg^–1^ and 783 Wh L^–1^) of NMC811|SE|CNT were increased compared to those of commercial Li-ion batteries, suggesting a readily deployable option for exploiting Li metal as an anode. Our method can be versatile in desirable Li plating with a stable SEI by delocalizing Li^+^ flux (with SE-containing separators) and introducing large surface areas for Li^+^ plating/stripping (with porous anodes). The effectiveness of this approach would increase with the Li^+^ conductivity of the SE material and the surface area available for distributing Li plating.

## Methods and Experimental Details

### SE Composite and CNT Anode Preparation

The SE composite was fabricated by blade casting a homogeneous slurry of poly(vinylidene fluoride-*co*-hexafluoropropylene (PVDF-HFP) (*M*_w_ ≈ 400000) and Li_6.4_La_3_Zr_1.4_Ta_0.6_O_12_ (LLZTO) (50:50 weight ratio) on a polypropylene (PP) separator. For the CNT anode, cylindrical spongelike porous CNT frameworks, which were synthesized through a chemical vapor deposition (CVD), were sliced and vacuum filtered with a solution composed of potassium permanganate (KMnO_4_) and sulfuric acid (H_2_SO_4_) to create trench-wall unzipped structures. After rinsing and drying, the CNT films were chopped into particles of a few hundred micrometers by utilizing high-energy ball milling. Finally, CNT particles were mixed with poly(vinylidene fluoride) (PVDF, *M*_w_ ≈ 534000) at a weight ratio of 95:5 in *N*-methyl-2-pyrrolidinone (NMP) and then cast on Cu foil to form the CNT electrode. Finally, the Li|CNT asymmetrical cells were assembled. After the lithiation cycles (voltage window of 0–2 V for 10 cycles) and Li plating cycles (voltage window of −0.5–0.5 V for 10 cycles), the CNT anode with a predetermined amount of Li was taken out and rinsed in 1,3-dioxolane (DOL) to remove salts and then dried for further experiments. Detailed procedures of SE composite and CNT anode preparation are described in the Supporting Information (preparation of SE composite separators, preparation of the 3D CNT framework and electrode, and preparation of the Li-deposited CNT anode).

### Electrolyte Fabrication

A carbonate-ester electrolyte was prepared by dissolving 1.0 M lithium hexafluorophosphate (LiPF_6_) (≥99.99%, Sigma-Aldrich) and 0.05 M lithium difluoro(oxalate)borate (LiDFOB) (95.0%, AmBeed) in a solution of ethyl methyl carbonate (EMC, 99.9%, Sigma-Aldrich) and 4-fluoro-1,3-dioxolan-2-one (fluoroethylene carbonate or FEC, >98.0%, TCI) with a volume ratio of 3:1.

### Cell Assembly and Testing Conditions

For Li symmetrical cells (Li|SE|Li, Li|PP|Li) for SEM and XPS, 65 μm thickness Li foils and PP or SE separators were assembled and tested by a Neware battery test system (BTS4000-5V10mA). For Li plating tests (Li|PP|CNT, Li|PP|Cu), 200 μm thick Li foil, and a CNT anode without predeposited Li or Cu foil were assembled and tested by an Arbin battery test system (LBT21084). For full-cell tests, the NMC811 (areal capacity of 2.0 mAh cm^–2^ or 4.0 mAh cm^–2^) and the LFP (areal capacity of 1.25 mAh cm^–2^) were assembled with different separator|anode pairs (e.g., PP|Li, PP|CNT, and SE|CNT). For the lean electrolyte conditions test, NMC811 cathodes with an areal capacity of 4.0 mAh cm^–2^ were assembled with 14 μL (4.3 g Ah^1–^) of the carbonate-ester electrolyte. The operando cell was fabricated with a pouch cell and a cover glass (thickness No. 1) as a viewing window. The current collector was made by wrapping the Cu foil around the glass slides, and then Li foil was placed on one side with the separator located on another side. After the cell was filled with 500 μL of the electrolyte, Li plating/stripping processes were carried out. Detailed information for all cell assembly and testing conditions are described in the Supporting Information (cell assembly and testing conditions, in-operando cell assembly).

### Characterizations

Detailed information regarding material characterizations, X-ray diffraction (XRD), Fourier transform infrared (FTIR) spectroscopy, optical microscopy, scanning electron microscopy (SEM), X-ray photoelectron spectroscopy (XPS), and electrochemical characterizations, linear sweep voltammetry (LSV), electrochemical impedance spectroscopy (EIS), and chronoamperometry (CA) polarization is described in the Supporting Information (material characterization, electrochemical measurements).

### Finite Element Analysis (FEA)

The migration behavior and concentration distribution of Li^+^ through different separator|anode pairs were analyzed by utilizing a finite element analysis tool (COMSOL Multiphysics 6.0). For PP|Li and SE|Li pairs, electrostatic and transport of diluted species models were selected. For PP|CNT and SE|CNT pairs, electrostatic and transport of diluted species in porous media model were selected. Detailed information such as simulation domain geometries, governing equations, global and local parameters, and boundary conditions to simulate the simplified Li^+^ migration under electrical potential and concentration gradients is minutely described in the Supporting Information (finite element analysis simulation methods).

## Supporting Information Available

Preparation of SE composite separators, preparation of the 3D CNT framework and electrode, preparation of Li-deposited CNT anode, cell assembly and testing conditions, in-operando cell assembly, material characterization, electrochemical measurements, finite element analysis simulation methods, energy density calculation for the NMC811|SE|CNT full cell and cell-level energy density calculations for the commercial Li-ion batteries, FEA analysis domain geometries and Li^+^ concentration, gradient, and flux distribution results, SEM images, XRD and FT-IR, Li^+^ transference number, LSV profiles, overpotential profiles while Li plating/stripping, voltage-capacity profiles for half cells and full cells, Nyquist plots, rate capability, and detailed information on the commercial Li-ion batteries in Figure 8b. (PDF)
Delocalized Li metal plating on a Cu current collector when the composite separator (Li|SE|Cu cell) is used (MP4).
Localized Li plating with a PP separator (Li|PP|Cu cell) (MP4).
