# Supplementary material for: Delocalized Lithium Ion Flux by Solid-State Electrolyte Composites Coupled with 3D Porous Nanostructures for Highly Stable Lithium Metal Batteries
Source: ACS Nano. 2023 Jul 29;17(16):16020–35. doi: 10.1021/acsnano.3c04526 (PMC10863402; doi:10.1021/acsnano.3c04526)
Supplement: Supplementary file 1 — nn3c04526_si_001.pdf [file nn3c04526_si_001.pdf]

SUPPORTING INFORMATION

Delocalized Lithium Ion Flux by Solid-State Electrolyte Composites Coupled with 3D Porous Nanostructures for Highly Stable Lithium Metal Batteries

Jooyoung Lee[1], Hyunji Park[1], Jieun Hwang[1], Juran Noh[2], Choongho Yu[1,2]*

[1] Department of Mechanical Engineering

[2] Department of Material Science and Engineering

Texas A&M University, College Station, Texas 77843 USA

*Corresponding author: chyu@tamu.edu

Experimental

Preparation of solid-state electrolyte (SE) composite separators

The SE composite layer was fabricated using the general blade casting method. First, 0.75 g of poly(vinylidene fluoride-co-hexafluoropropylene (PVDF-HFP) (M_w~400,000, Sigma Aldrich) pellets were dissolved into 4 g of acetone solution, then the mixture was magnetically stirred at 50 ℃ for 6 hours. Subsequently, 0.16 g of $Li_{6.4}La_3Zr_{1.4}Ta_{0.6}O_{12}$ (LLZTO) (> 99.9%, Ampcera Inc.) powders were dispersed in the 1 g of the mixture solution with the weight ratios (LLZTO : PVDF-HFP) in the main manuscript with continuous stirring for additional 6 hours at room temperature. The final homogeneous slurry of PVDF-HFP and LLZTO was cast on a conventional polypropylene (PP) separator (Celgard 2500, 55% porosity according to the specification) using a doctor blade with a gap height of 50 μm. The membrane was then immediately transferred to a vacuum oven and dried under 30 ℃ for 12 hours to completely evaporate the solvent. Finally, the fully-dried membrane was punched to have coin shape separators whose diameters are 15.88 mm (5/8 inch).

Preparation of the 3D carbon nanotube (CNT) framework and electrode

Cylindrical sponge-like porous CNT frameworks were synthesized by a chemical vapor deposition (CVD) process. The detailed CVD procedure is described in our previous study.[S1] The cylindrical CNT was sliced into films (100~200 μm) using a razor blade. Then, a mechano-chemical treatment of the sliced CNT was carried out using a vacuum filtration set up with a solution composed of $KMnO_4$ (> 99 %, AMRESCO) and sulfuric acid (95~98%, BDH Chemicals). This acid treatment creates trench-walls and carboxyl functional groups on CNT, as explained in our paper.[S2] After completing the acid treatment, the sliced CNT films were rinsed with deionized water, and then fully dried under 60 ℃ for 12 hours. The CNT films were chopped into pieces using a high-energy ball miller (SPEX SamplePrep 8000M Mixer Mill) with a hardened steel container (SPEX SamplePrep 8001) with two chrome steel balls (5 mm diameter,

Swordfish). CNT particles whose diameters are a few hundred microns were selectively collected using sieves. The CNT particles were mixed with polyvinylidene fluoride (PVDF, M_w ~534,000, Sigma Aldrich) at a weight ratio (CNT:PVDF) of 95:5 in N-methyl-2-pyrrolidinone (NMP, > 99%, Sigma Aldrich) with a solid-to-solvent ratio of 100 mg/mL using a mortar and pestle for 5 minutes. The slurry was coated on a Cu foil using a doctor blade with a gap height of 450 microns, and fully dried in a vacuum oven under 60 ℃ for 12 hours. The CNT electrode was cut into circular shapes with a diameter of 9.5 mm (3/8 inch).

Preparation of Li-deposited CNT anode

Li|CNT asymmetric cells were assembled using the prepared 3D CNT electrode and Li metal (200 μm in thickness, 99.9%, Alfa Aesar) with a CR2032 coin cell in an Ar-filled glovebox (O_2 < 0.5 ppm, H_2O < 0.5 ppm). The electrolyte was prepared by dissolving 1.0 M lithium bis(trifluoromethanesulfonyl)imide (LiTFSI) (99.0 %, Sigma Aldrich) and 0.5 M lithium nitrate ($LiNO_3$) (99.0%, Alfa Aesar) in a mixture of 1,3-dioxolane (DOL) (99+%, Alfa Aesar) and 1,2-dimethoxyethane (DME) (99+%, Alfa Aesar) (volumetric ratio of 1:1). The assembled cell was initially cycled at 0.5 mA cm^{-2} with a cutoff voltage window of 0~2 V for 10 cycles for fully lithiating the CNT.[S3] Thereafter, a predetermined amount of Li (6 mAh cm^{-2} for CNT anode of the Li metal plating tests in Fig.6c, 10 mAh cm^{-2} for CNT anode of the NMC811|SE|CNT, NMC811|PP|CNT, LFP|SE|CNT, and LFP|PP|CNT full cells) was plated into the pore of the CNT at 1.0 mA cm^{-2} until a predetermined capacity (mAh cm^{-2}) and a lower cut-off voltage of -0.5 V. After finishing the first lithium metal deposition process, 10 more stripping/plating cycles were carried out at the same current density and capacity conditions with a voltage window of -0.5~0.5 V. The Li-deposited CNT electrode was taken out of the Li|CNT cell, and rinsed in DOL to remove salts and then dried for further experiments.

Cell assembly and testing conditions

A carbonate-ester based electrolyte was prepared by dissolving 1.0 M lithium hexafluorophosphate ($LiPF_6$) (≥ 99.99%, Sigma Aldrich) and 0.05 M lithium difluoro(oxalate)borate (LiDFOB) (95.0%, AmBeed) in a solution of ethyl methyl carbonate (EMC, 99.9%, Sigma Aldrich) and 4-fluoro-1, 3-dioxolan-2-one (fluoroethylene carbonate or FEC, > 98.0 %, TCI) with a volume ratio of 3:1. LiDFOB was used as an electrochemical reduction agent in the EMC/FEC electrolyte to have an inorganic Li-F and Li_2CO_3 rich SEI layer,[S4–S7] which can induce uniform lithium growth and thereby suppress dendrite growth.[S8–S10]

Li|SE|Li and Li|PP|Li symmetric cells for SEM and XPS characterization were assembled using 2032 coin cells with a Li metal foil (Rockwood, battery grade) whose thickness is 65 μm (total areal capacity ~13.4 mAh cm^{-2}) and diameter is 9.5 mm (3/8 inch). All the PP separators used in this study are Celgard 2500 whose thickness is ~25 μm. The symmetric cells were tested using a Neware battery test instrument (BTS4000-5V10mA) with a current density of 1.0 mA cm^{-2} and a plating/stripping capacity of 2.0 mAh cm^{-2}. The amount of the carbonate-ester electrolyte used was 50 μL per cell. All the coin cell testing was carried out using electrodes whose diameters are 9.5 mm (3/8 inch) and separators whose diameters are 15.9 mm (5/8 inch).

The Li metal plating tests for Li|PP|CNT and Li|PP|Cu asymmetric cells were conducted using 2025 coin cells with the 200-μm thick lithium foil (total areal capacity ~41.2 mAh cm^{-2}) and the 3D CNT electrode without Li metal deposition or a Cu foil (9 μm in thickness) under a constant current (1.0 mA cm^{-2}) discharge condition with a lower cut-off voltage of -0.5 V using an Arbin test instrument (LBT21084). The amount of the carbonate-ester electrolyte used was 50 μL per cell. The EIS measurement for both cells was conducted using a Gamry instrument (Interface 1010 E).

Li|SE|CNT asymmetric and Li|PP|Li symmetric cells (Fig. 6c, Fig.S16, Fig.S17) employed the 65-μm thick Li foil as a cathode, the SE composite or PP membrane as a separator, and the 3D CNT or Cu foil with Li deposition of 6.0 mAh cm^{-2} as an anode. The Neware battery test instrument was used to supply

current densities of 0.5 mA cm^{-2} or 1.0 mA cm^{-2} and a capacity of 2.0 mAh cm^{-2}. The amount of the carbonate-ester electrolyte used was 50 µL per cell.

For the full cell tests, the NMC811 cathodes (areal capacities of 2.0 mAh cm^{-2} and 4.0 mAh cm^{-2}, respectively) and the LFP cathode (areal capacity of 1.25 mAh cm^{-2}) were purchased from NEI Corporation. According to the specification sheet, the NMC811 cathode with an areal capacity of 2.0 mAh cm^{-2} (NANOMYTE BE-56E, NEI Corp.) has a total loading of 10.64 mg cm^{-2} and a total active material loading of 9.58 mg cm^{-2} ($\pm$ 0.12 mg cm^{-2}). The NMC811 cathode with an areal capacity of 4.0 mAh cm^{-2} (Custom-made NANOMUTE BE-56E, NEI Corp.) has a total loading of 23.18 mg cm^{-2} and the total active material loading of 20.86 mg cm^{-2} ($\pm$ 0.2 mg cm^{-2}). The LFP cathode (NANOMYTE BE-60E) has the total loading of 8.47 mg cm^{-2} and the total active loading of 7.62 mg cm^{-2} ($\pm$ 0.07 mg cm^{-2}). All the full cells were assembled using 2025 coin cells. The NMC811 and LFP full cells were conducted with voltage windows of 2.8~4.3 V and 2.5~4.1 V (vs. Li/Li^+), respectively, at room temperature. The values of 1 C rate for NMC811 and LFP were set as 200 and 170 mAh g^{-1}, respectively. The amount of the carbonate-ester electrolyte used for all the full cell tests was 40 µL per cell.

For the full cell test under the lean electrolyte condition (Fig. 8a,b), NMC811 cathodes with an areal capacity of 4.0 mAh cm^{-2} were assembled using 2025 coin cells. The amount of the carbonate-ester electrolyte used for this test was 14 µL (4.3 g Ah^{-1}) per cell. The voltage window range was 2.8-4.3 V and the value of 1 C rate was set as 200 mAh g^{-1}.

In-Operando cell assembly

The in-operando cell was fabricated with a pouch cell and a cover glass (thickness No.1) as a viewing window. The current collector was made by wrapping the Cu foil around microscope glass slides whose thickness is 1.0 mm. Li metal (99.9%, Alfa Aesar) was placed at one side of the Cu current collectors, and the separator was placed between the two electrodes. Then the two Cu/glass current collectors were

pushed against each other. The pouch cell was filled with 500-μL electrolyte (1 M LiTFSI and 0.5 M $LiNO_3$ in a mixture of DOL and DME (1:1 by vol.)). All the lithium metal deposition processes were carried out at a current density of 4.0 mA cm^{-2} and a capacity of 4.0 mAh cm^{-2}. The dark-field optical microscope (Olympus BX5) images were taken every 1 minute using Q capture Pro 6.0 software and the obtained images were integrated into video files (Supplementary Movie 1 and 2) by using the Windows Pictures application.

Material characterization

The X-ray diffraction (XRD) patterns were obtained by utilizing a BRUKER D8 equipment with a scan range (2θ) from 10º to 80º with a 0.05º sec^{-1} scanning speed at room temperature. The Fourier transform infrared (FTIR) spectroscopy analysis was performed using Thermo Nicolet 380 FTIR spectrometer between 4000 and 500 cm^{-1} with a wavenumber resolution of 1 cm^{-1} at room temperature. The dark-field optical microscope (Olympus BX5) images were taken using Q capture Pro 6.0 software. The SEM images were taken with a JEOL JSM-7500F filed-emission scanning electron microscope. The X-ray photoelectron spectroscopy (XPS) was carried out with an Omicron XPS system with DAR 400 dual Mg/Al X-ray source ($< 5 \times 10^{-9}$ Torr condition).

Electrochemical measurements

The electrochemical oxidation stability of the separators was evaluated with a Li|separator|Li symmetric cell configuration by linear sweep voltammetry (LSV) at a scan rate of 5 mV s^{-1} with a voltage window of 0~8 V. The ionic conductivity (σ) was obtained by the following equation:

$$\sigma = \frac{t}{R_b \times A}\ [mS\ cm^{-1}] \quad [\text{Eq. S1}]$$

where R_b is a bulk impedance, t is the thickness of the separator, and A is a contact area between the separator and stainless steel (SS) current collector. The bulk impedance (R_b) was measured by electrochemical impedance spectroscopy (EIS) in a symmetric SS|separator|SS cell over a frequency range of 10^5~0.1 Hz.

The Li^+ transference numbers (t_{Li+}) of the different separators were obtained from the chronoamperometry (CA) polarization and EIS before and after the polarization. EIS was recorded from 0.1 Hz to 100 kHz. CA was tested at a static potential (ΔV) of 10 mV for 5,000 sec. The galvanostatic polarization process was performed using the Arbin battery tester. The following equation was used to calculate t_{Li+}

$$t_{Li^+} = \frac{I_{ss}(\Delta V - I_0 R_0)}{I_0(\Delta V - I_{ss} R_{ss})} \quad [\text{Eq. S2}]$$

where I_0 and I_{ss} are the initial and steady-state current, respectively. R_0 and R_{ss} are the electrolyte/electrode contact impedance measured before and after polarization, respectively, which were measured by EIS with an AC voltage of 5 mV after the five plating/stripping cycles at 1.0 mA cm^{-2} and a 2.0 mAh cm^{-2}. The equivalent impedance circuit for the fitting is shown below.

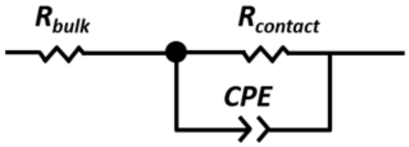

where R_{bulk} is the bulk impedance; $R_{contact}$ is the electrolyte/electrode contact impedance; and CPE is a constant phase element. R_0 and R_{SS} were obtained from the x-intercepts of the fitted Nyquist plot.

Finite element analysis simulation methods

Finite Element Analysis (FEA) software (COMSOL Multiphysics 6.0) was used to analyze the migration and the concentration distribution of Li^+ through different separator-anode pairs (PP|Li, SE|Li, PP|CNT, and SE|CNT). The geometrical domain structures of each case are represented in Fig.S1. Electrostatic and Transport of diluted species models were selected and coupled to simulate the simplified Li^+ migration under an electrical potential gradient through PP and SE separators for both PP|Li and SE|Li pairs. Governing equations of the coupled physics are represented in Equation S3-S5

$$\boldsymbol{E} = -\nabla V \quad \text{[Eq. S3]}$$

$$\boldsymbol{J} = -D\nabla c + z\mu Fc\boldsymbol{E} \quad \text{[Eq. S4]}$$

$$\frac{\partial \mathrm{c}}{\partial \mathrm{t}} = -\nabla \boldsymbol{J} \quad \text{[Eq. S5]}$$

Where $\boldsymbol{E}$ is the electric field (V), V is the electric potential (V), $\boldsymbol{J}$ is the flux vector of Li^+ (mol m^{-2} s^{-2}), D is the diffusion coefficient of Li^+ (m^2 s^{-1}), c is Li^+ concentration (mol m^{-3}), z is the charge number of Li^+, and F is a faraday constant.

For PP|CNT and SE|CNT pairs, Electrostatic model and Transport of diluted species in porous media model were coupled to simulate the Li^+ migration. Equation S3 was used to define the electric potential through the system as same as in PP|Li and SE|Li case. To simulate the Li^+ migration through the porous CNT domain, Equation S4 and Equation S5 becomes Equation S6 and Equation S7, respectively.

$$\boldsymbol{J} = -D_e\nabla c + z\mu Fc\boldsymbol{E} \quad \text{[Eq. S6]}$$

$$\frac{\partial \varepsilon_p \mathrm{c}}{\partial \mathrm{t}} = -\nabla \boldsymbol{J} \quad \text{[Eq. S7]}$$

$$D_e = \frac{\varepsilon_p}{\tau_F} D_F \quad \text{[Eq. S8]}$$

$$\tau_F = \varepsilon_p^{-1/3} \quad \text{[Eq. S9]}$$

Where D_e in Equation S8 is an effective diffusion coefficient (m^2 s^{-1}), ε_p in Equation S9 is a porosity of the CNT, τ_F is Milington and Quirk model coefficient for calculating the effective diffusivity, and D_F is a diffusion coefficient of the CNT (m^2 s^{-1}). The geometrical domains to which the governing equations are applied were set as rectangular areas with a size of 8.8 μm by 35.0 μm (PP|Li, PP|CNT) and 8.8 μm by 50.0 μm (SE|Li, SE|CNT), respectively. PP separator was designed as a 25 μm thick rectangular geometry composed of five rectangular pores with different widths (1.32, 1.0, 0.88, 0.7, and 0.5 μm, respectively), which are completely filled with a liquid electrolyte, and uniform pore spacing (0.88 μm) to simulate the non-uniform pores on the surface. SE composite separator was designed by attaching a simplified PVDF-HFP/LLZTO composite layer below the PP separator by referring to the SEM image (Fig. 3b). CNT domain was designed as a corrugated feature with uniform 0.2 μm intervals between each space by referring to the SEM image of the slurry cast 3D CNT framework (Fig. S5). The spacing between the top and bottom boundaries and the separator was set to 5.0 um to investigate the concentration distribution in the vicinity of the bottom electrode surface. For the top surface boundary, the Dirichlet boundaries with V = 20 mV and c = 1.0 M were applied. For the bottom surface boundary, the Dirichlet boundaries with V = 0 V and c = 0 M were used to generate electrical potential difference and concentration gradient between two electrodes. The other boundaries were set as natural boundaries with zero flux conditions. The diffusion coefficients of liquid electrolyte[S11], LLZTO particles[S11], and CNT framework[S12] were selected as 3.0 E-10, 6.0 E-12, and 1.0 E-11, respectively according to the previously demonstrated reference results. The porosity (ε_p) of the CNT structure was set as 0.3 by referring the theoretically calculated porosity of CNT anode with pre-deposited 10 mAh cm^{-2} of Li metal. Li^+ mobility (μ) for liquid electrolyte and LLZTO particles were defiend by the Nernst-Einsten equation. Li^+ concentration gradients in the LLZTO particle domain are present with a diffusion coefficient of 6.0 E-12 m^2 s^{-1} in the simulation, as shown in Figure S3. The concentration gradient ranged from is about 85000 to about 95000 mol m^{-4} within LLZTO domain. This internal Li^+ concentration gradient may lead to additional Li^+ migration through LLZTO. However, Li^+ flux (Fig. S4) through LLZTO (on average 6.0E-7 mol $m^{-2}s^{-1}$) is much smaller than that through the liquid electrolyte

domain due to the low diffusivity of LLZTO compared to the liquid electrolyte. This implies that the major Li^+ diffusion in the SE separator occurs through the pores inside the membrane which is filled with the electrolyte. The normalized Li^+ concentration near the anode surface for each separator-anode pair were measured and calculated through the line which is placed under 100nm from the bottom of the separator (gray dotted line in Fig. 2b-c). Since the purpose of the simulation in this work focused on comparing Li^+ migration propensity in the presence or absence of nanoporous structure of the SE separator and the normalized Li^+ concentrations of each case were measured below the separator region (not at the surface with Dirichlet boundary condition), Faradaic reaction and Li plating behavior were not considered this work.

Energy density calculation for the NMC811|SE|CNT full cell

To calculate the cell-level gravimetric energy density (E_g) of the NMC811|SE|CNT full cell, the following relations were used.

$$E_g = \frac{E_{full\ cell}}{m_{electrode} + m_{sep.} + m_{Al.cc} + m_{Cu.cc} + m_{electrolyte}}$$

$$= \frac{V_{avg} \cdot m_{AM} \cdot C_s}{m_{anode} + m_{cathode} + m_{sep.} + m_{Al.cc} + m_{Cu.cc} + m_{electrolyte}} \quad \text{[Eq. S10]}$$

E_g: Gravimetric energy density (Wh kg^{-1})

$E_{full\ cell}$: Energy of the full cell (Wh)

V_{avg}: Theoretical discharge voltage (for the NMC811 = 3.85 V[S13])

m_{AM}: Active material loading of the cathode (for the NMC811 = 20.86 mg cm^{-2})

C_s: Specific capacity of the full cell (mAh g^{-1})

$m_{electrode}$: Areal mass of the cathode and anode (mg cm^{-2})

$m_{sep.}$: Areal mass of the separator (mg cm^{-2})

$m_{Al.cc}$: Areal mass of the aluminum current collector (mg cm^{-2})

$m_{Cu.cc}$: Areal mass of the copper current collector (mg cm^{-2})

m_{anode}: Areal mass of the anode (mg cm^{-2})

$m_{cathode}$: Areal mass of the cathode (mg cm^{-2})

$m_{electrolyte}$: Areal mass of the carbonate-ester electrolyte (mg cm^{-2})

To calculate the maximum gravimetric energy density of the NMC811|SE|CNT (Fig. 8b), V_{avg} = 3.85 V, m_{AM} = 20.86 mg cm^{-2}, the maximum C_s = 201.2 mAh g^{-1} at 34th cycle, $m_{cathode}$ of the high loading NMC811 (4.0 mAh cm^{-2}). The total mass (23.18 mg cm^{-2}) of the cathode consists of 90% active material, 5% PVDF binder, and 5% Super P according to the manufacturer's specification.

The areal mass of the Li deposited CNT anode (m_{anode}) was obtained using the following relations.

$$m_{anode} = m_{CNT} + m_{Li} = m_{CNT} + (C_{Li\ depo.}/C_{s\ Li\ theor.})$$

m_{CNT}: Areal mass of the 3D CNT framework.

m_{Li}: Areal mass of the deposited lithium metal in the 3D CNT framework.

$C_{Li\ depo.}$: Areal capacity of the deposited Li (mAh cm^{-2}).

$C_{s\ Li\ theor.}$: Specific theoretical capacity of Li metal (mAh g^{-1}).

For NMC811|SE|CNT full cell with the high loading NMC811 (4.0 mAh cm^{-2}), 80-µm thick CNT framework was used, and the areal mass was 1.92 mg cm^{-2}. To have the N/P ratio of 2.5, Li metal with a capacity ($C_{Li\ depo.}$) of 10 mAh cm^{-2} was deposited into the CNT framework. By dividing the theoretical

capacity of Li metal ($C_{s\,Li\,theor.}$ = 3860 mAh g^{-1}), $C_{Li\,depo.}/C_{s\,Li\,theor.}$, the areal mass of the deposited lithium metal (m_{Li}) was obtained. Therefore, for the amount of 10 mAh cm^{-2}, m_{Li} was calculated to be 2.59 mg cm^{-2}. The total mass of the anode including the Li metal and CNT (m_{anode}) was ~4.5 mg cm^{-2}.

The areal mass of the SE composite separator (total thickness of 40 microns) was calculated to be 2.07 mg cm^{-2} based on the theoretical densities of solid-state electrolyte (SE), PVDF-HFP, and PP. Likewise, $m_{Al.cc}$ and $m_{Cu.cc}$ were calculated to be 2.03 mg cm^{-2} and 4.03 mg cm^{-2}, respectively, where the effective thicknesses of Al and Cu are 7.5 μm and 4.5 μm, respectively.

The areal mass of the carbonate-ester electrolyte was calculated by applying the calculated theoretical density of the 1M $LIPF_6$ and 0.05M LIDFOB in the EMC and FEC with a volume ratio of 3:1 electrolyte (1.29 g cm^{-3}). To calculate the gravimetric cell-level energy density, the following two assumptions were considered. First, the entire pore volume of the cathode, anode, and separator are filled with electrolyte. According to the porosity, area, and thickness of the electrodes and separator, the total areal volume of the electrolyte to fill the pore was calculated as 0.00698 cc cm^{-2} (0.00218554 cc cm^{-2} for the cathode, 0.002219 cc cm^{-2} for the anode, and 0.002575 cc cm^{-2} for the separator). Therefore, the areal mass of the electrolyte (9 mg cm^{-2}) to fill the pore was achieved by multiplying the density and the areal volume. By considering the area of each component (1.2667 cm^2 for the cathode and the anode, and 1.979 cm^2 for the separator), the total volume of the electrolyte to fill the pore was calculated to be 10.67 µL. The 3.33 µL of the excess electrolyte was added to the system since we used 14 µL in the full cell. Note that we did not have the formation process (which is typical in fabricating commercial cells). Therefore, some of the electrolyte should have been used to form the SEI layer. The areal mass of the extra amount of the electrolyte (3.33 µL) was calculated as 3.39 mg cm^{-2} by considering the density of the electrolyte (1.29 g cm^{-3}) and the area of the electrode (1.2667 cm^{-2}). Therefore, the total areal mass of the carbonate-ester electrolyte ($m_{electrolyte}$) was calculated to be 12.39 mg cm^{-2}.

According to the aforementioned procedure, the maximum gravimetric energy density (Wh kg^{-1}) of the NMC811|SE|CNT full cell was obtained as follows.

$$E_g = \frac{V_{avg} \cdot m_{AM} \cdot C_s}{m_{anode} + m_{cathode} + m_{sep.} + m_{Al.cc} + m_{Cu.cc} + m_{electrolyte}} =$$

$$\frac{3.85\ [V] \cdot 20.86\ [mg/cm^{-2}] \cdot 201.2\ [mAh/g]}{4.5\ [mg/cm^{-2}] + 23.33\ [mg/cm^{-2}] + 2.07\ [mg/cm^{-2}] + 2.03\ [mg/cm^{-2}] + 4.03\ [mg/cm^{-2}] + 12.39\ [mg/cm^{-2}]} =$$

$$\sim 334\ [Wh/kg]$$

To calculate the maximum volumetric energy density (E_v) of the NMC811|SE|CNT full cell, the following relations were used.

$$E_v = \frac{E_{full\ cell}}{\forall_{total}} = \frac{V_d \cdot m_{AM} \cdot C_s}{t_{anode} + t_{cathode} + t_{Al,Cu,Separator}} \quad \text{[Eq. S11]}$$

E_v: Gravimetric energy density (Wh kg^{-1})

$E_{full\ cell}$: Energy of the full cell (Wh)

V_d: Nominal discharge voltage (for the NMC811 = 3.85 V)

m_{AM}: Active material loading of the cathode (for the NMC811 = 20.86 mg cm^{-2})

C_s: Specific capacity of the full cell (mAh g^{-1})

$\forall_{total}$: Total volume of the full cell including anode, cathode, current collectors, and separator (L)

t_{anode}: Thickness of the anode (For the NMC811|SE|CNT, total thickness of the CNT anode, μm)

$t_{cathode}$: Thickness of the cathode (NMC811 layer with a 4.0 mAh cm^{-2} areal capacity, μm)

$t_{Al,Cu,Separator}$: Summation of the thickness of the separator (SE composite separator, 40 μm), the effective thickness of the Al current collector at the cathode side (7.5 μm), and the effective thickness of the Cu current collector at the anode side (4.5 μm).

To calculate the maximum volumetric energy density of the NMC811|SE|CNT (Fig. 8b), V_d = 3.85 V, m_{AM} = 20.86 mg cm^{-2}, the maximum C_s = 201.2 mAh g^{-1} at 40th cycle, t_{anode} of the CNT anode with the 10 mAh cm^{-2} amount of the lithium metal deposited was measured to be 80 µm. t_{anode} was measured immediately after completing the lithium metal deposition process in the Ar-filled glove box using a micrometer. $t_{cathode}$ was measured to be 74.46 µm, and $t_{Al,Cu,Separator}$ was 52.0 µm. Then, the maximum volumetric energy density (Wh L^{-1}) of the NMC811|SE|CNT full cell was obtained as follows.

$$E_v = \frac{V_d \cdot m_{AM} \cdot C_s}{t_{anode} + t_{cathode} + t_{Al,Cu,Separator}}$$

$$= \frac{3.85\ [\mathrm{V}] \cdot 20.86\ [\mathrm{mg/cm^{-2}}] \cdot 201.2\ [\mathrm{mAh/g}]}{80\ [\mathrm{\mu m}] + 74.46\ [\mathrm{\mu m}] + 52\ [\mathrm{\mu m}]} = {\sim}783\ [\mathrm{Wh\ L^{-1}}]$$

Cell-level energy density calculations for the commercial Li-ion batteries

The cell-level gravimetric energy density values (including cathode, anode, separator, electrolyte, and current collectors) of the commercial Li-ion batteries consisting of graphite anodes paired with $LiNi_{1-x-y}Co_xMn_yO_2$ (NMC) or $LiMn_2O_4$-$LiNi_{1-x-y}Co_xMn_yO_2$ (LMO-NMC) cathode are shown in Fig. 8b and Table S7. Their specific energy (Wh kg^{-1}), capacity (Ah), and discharge voltage (V) data are available in the literature.[S14–S17] The weight fractions occupied by each component are 41% for the cathode, 18% for the anode, 4% for the aluminum current collector, 7% for the copper current collector, 17% for the housing, 3% for the separator, and 10% for the electrolyte).[S18] As for the cell-level gravimetric energy density of the commercial cells were calculated by dividing the specific energy density with 0.83 (83%) considering all of the components except the housing. For the volumetric energy density of the commercial cells, we assumed that the external housing is thin enough to neglect the volume of the casing, and the volumetric energy density results from the literature were cited without modification.[S17]

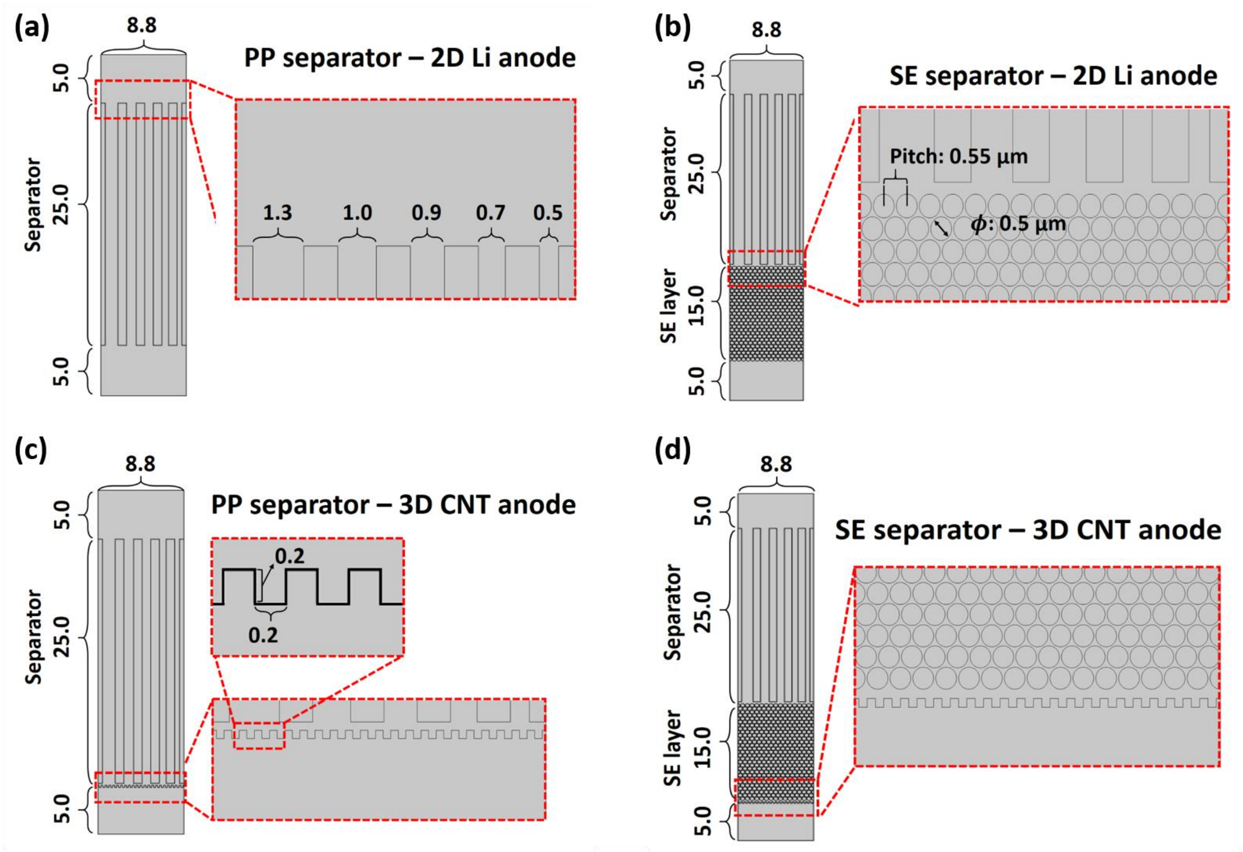

Figure S1. FEA simulation geometries for (a) PP|Li pair, (b) SE|Li pair, (c) PP|CNT pair, and (d) SE|CNT pair. Length unit for each figure is µm.

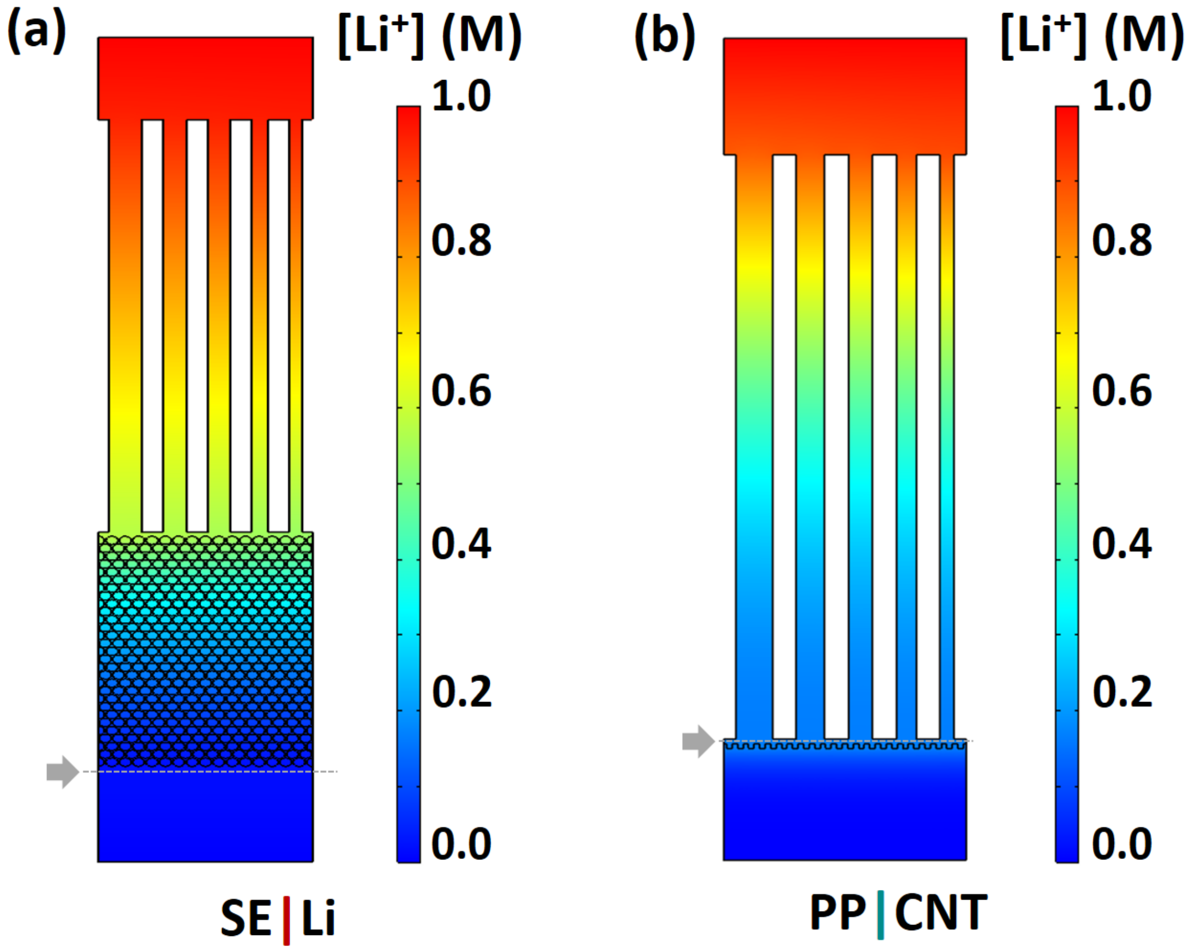

Figure S2. Simulation results of Li^{+} concentration distribution through (a) SE|Li and (b) PP|CNT separator|anode pair.

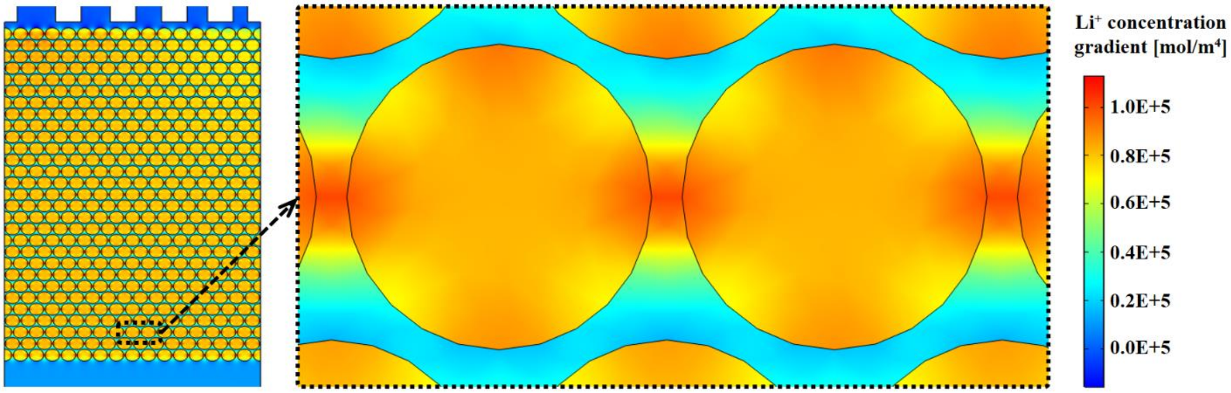

Figure S3. Li^+ concentration gradient distribution of the SE domain in the SE|Li pair. A rectangular portion containing two LLZTO particles was selected to show the details, indicating the average concentration gradient within LLZTO is about 90000 mol m^{-4}.

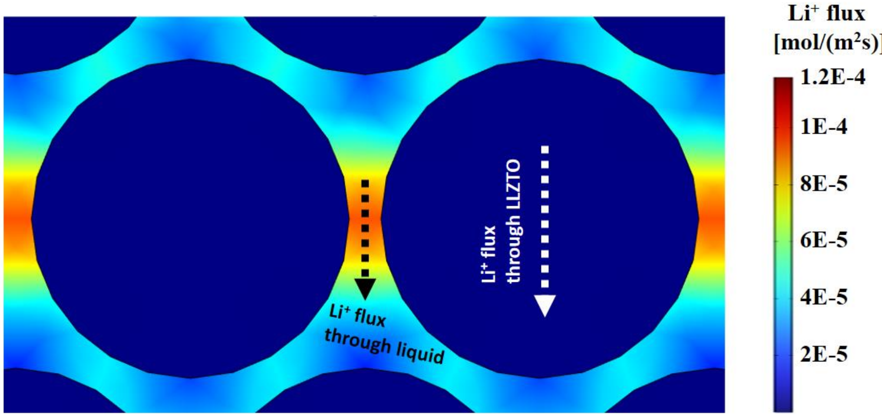

Figure S4. Li^+ flux (mol $m^{-2}s^{-1}$) distribution in the LLZTO and liquid electrolyte domain. The white dotted and black dotted arrows indicate the main direction of the flux through each domain.

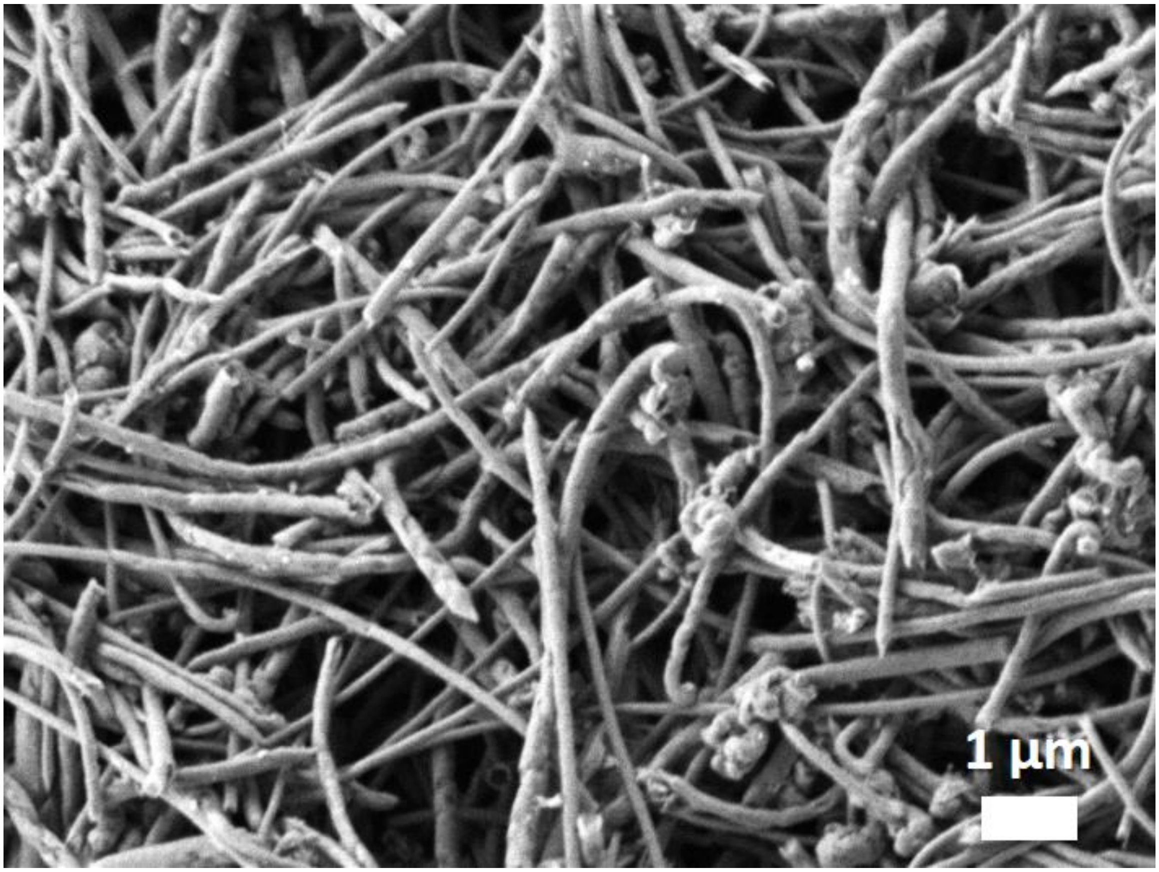

Figure S5. Top-view SEM image of the 3D CNT framework

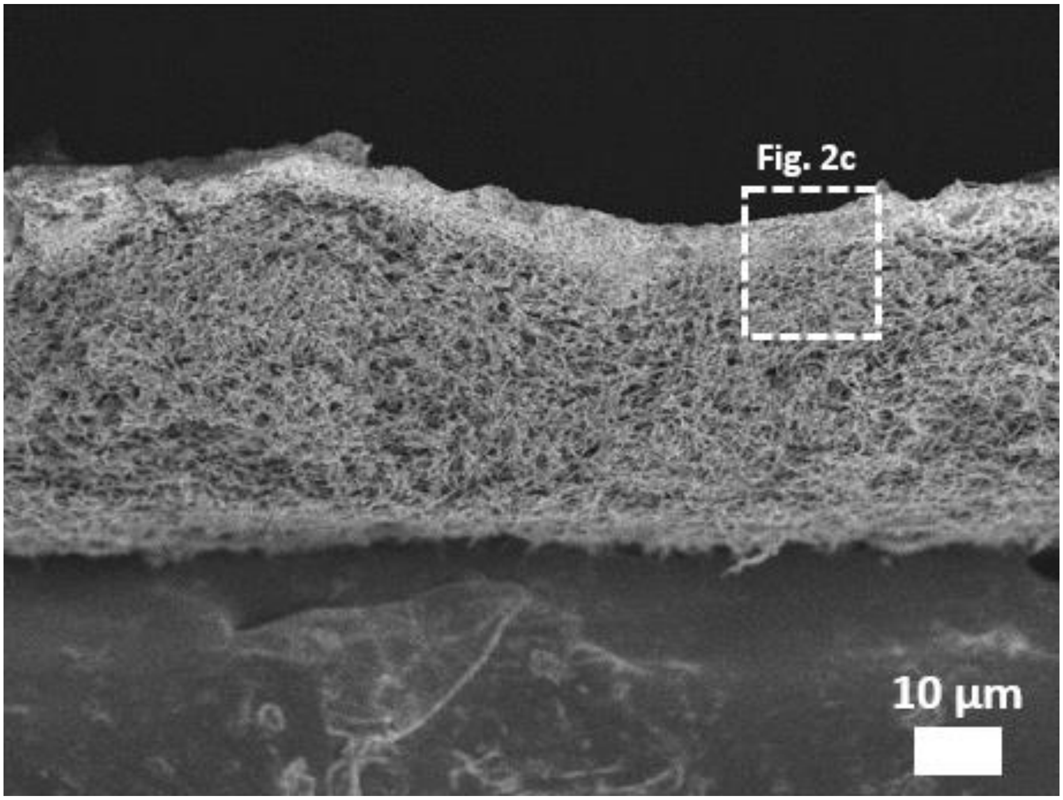

Figure S6. A low-magnification SEM image of the3D CNT anode with the SE composite layer on top. An enlarged image near the rectangular inset is shown in Fig. 3c.

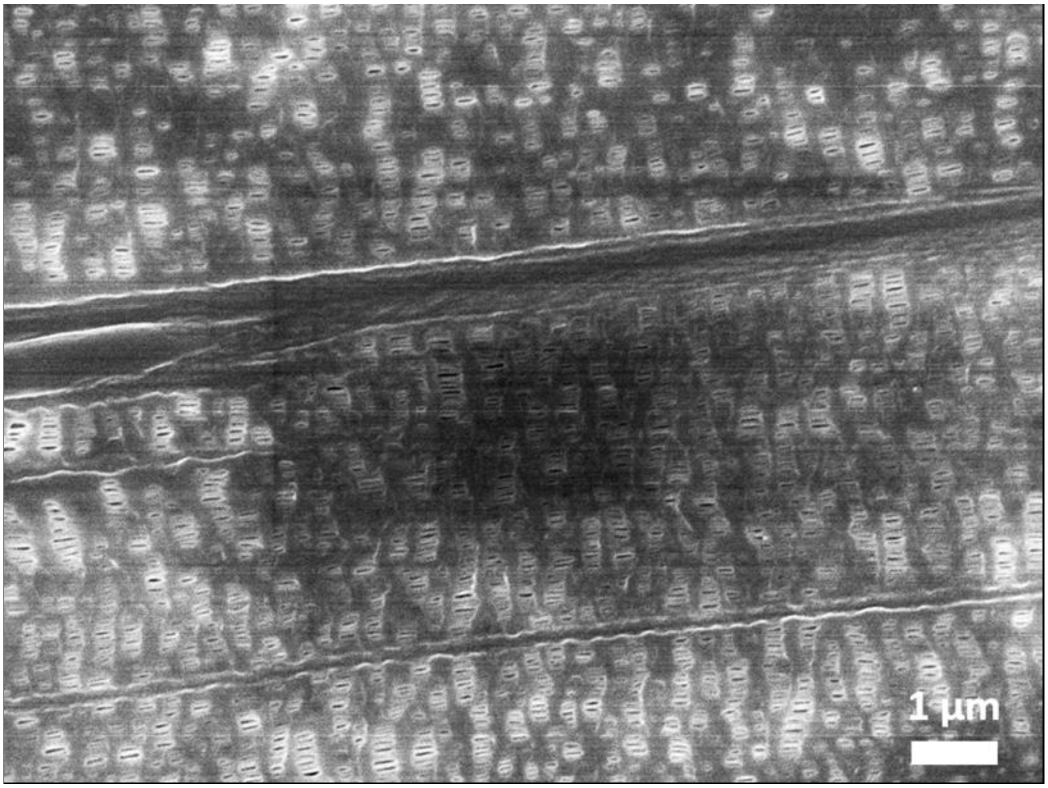

Figure S7. A SEM image of the surface of a commercial PP separator, showing non-uniformly distributed pores.

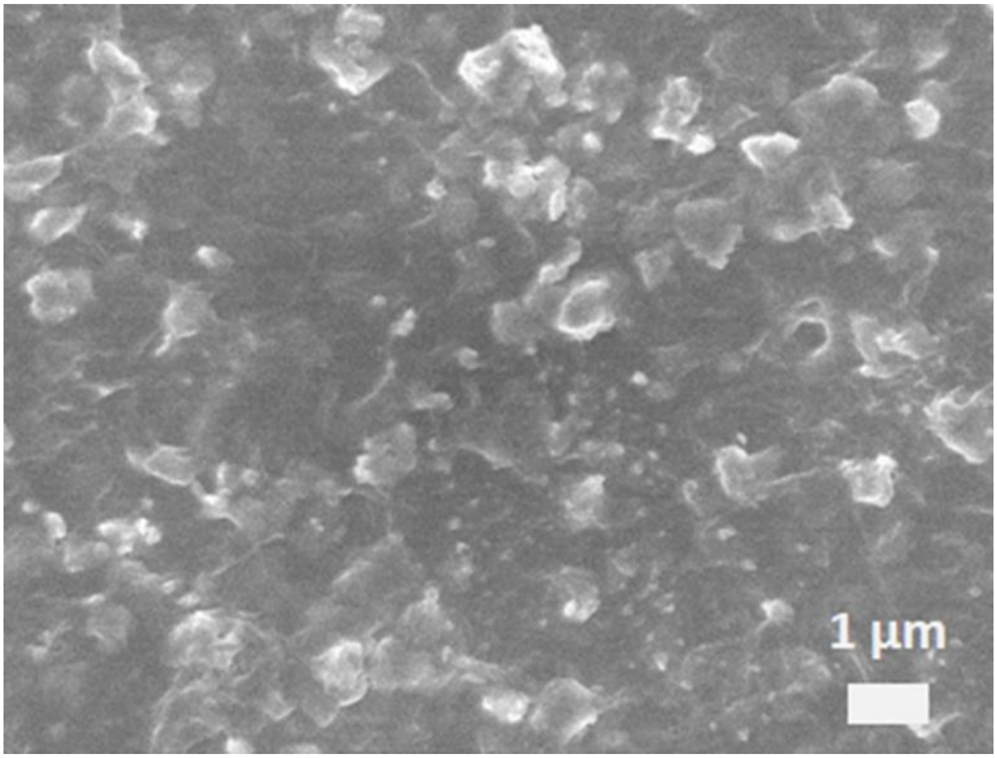

Figure S8. A SEM image of the surface of SE composite layer whose LLZTO concentration is 50 wt.%..

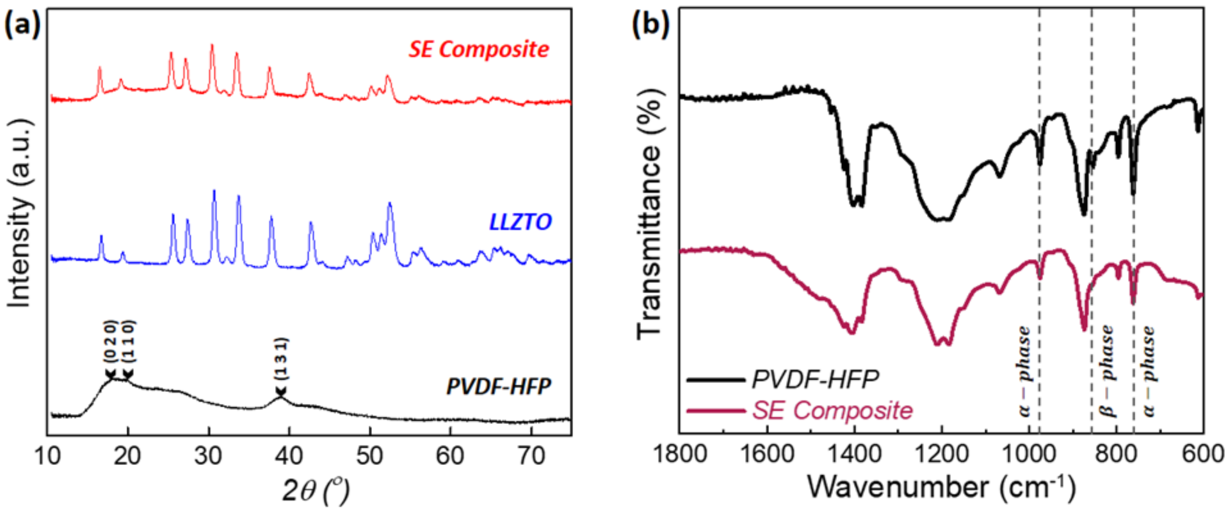

Figure S9. (a) XRD patterns of SE/polymer composite film (50 wt.% LLZTO), LLZTO powders (blue), and pure PVDF-HFP film (black). The pure PVDF-HFP had broad crystalline X-ray diffraction (XRD) peaks at around 20° ((0 2 0) at 18.5°[S19] and (1 1 0) at 20.2°[S20]) and 40° ((1 3 1) at 38.9°[S21]), which are consistent with the literature. In the SE/polymer composite, no additional crystalline peaks were detected, and the peaks were consistent with those of LLZTO. This suggests that significant chemical reaction that may alter their properties did not occur between LLZTO and PVDF-HFP. (b) FTIR spectra of pure PVDF-HFP and SE/polymer composite. The pure PVDF-HFP shows several absorption peaks at around 1402 cm^{-1}, 1179 cm^{-1}, and 1069 cm^{-1}, corresponding to CH_2 wagging [ω(CH_2)], anti-symmetric –CF_2 stretching [v_a(CF_2)], and CF_3 out of plane (rocking) deformation [γ(CF_3)],[S20, S22] respectively. Moreover, the lattice vibration of some representative crystalline phase structures of PVDF-HFP is also shown. For instance, at ~976 cm^{-1} and ~761 cm^{-1}, which correspond to the α-phase crystalline structure lattice vibration peaks were detected.[S23] In addition, at ~842 cm^{-1}, the β-phase peak of PVDF-HFP was detected.[S23] In the FTIR of the SE composite also exhibited the aforementioned peaks, suggesting the composite retains its stable phase during the synthesis process (no undesirable chemical reactions between LLZTO and PVDF-HFP). In addition, the crystalline peak intensities of the SE composite layer were weakened by adding LLZTO. Specifically, the peak intensities of the SE composite compared to pure PVDF-HFP corresponding to α-phase and β-phase crystalline structures diminished by 27 % and 45 %, respectively, when the crystalline peak intensities were normalized by the characteristic anti-symmetric –CF_2 stretching [v_a(CF_2), 1179 cm^{-1}] peaks. The above results elucidate that the addition of LLZTO to PVDF-HFP decreased the crystallinity of PVDF-HFP, which would have increased the ionic conductivity (σ) of the SE composite separator.

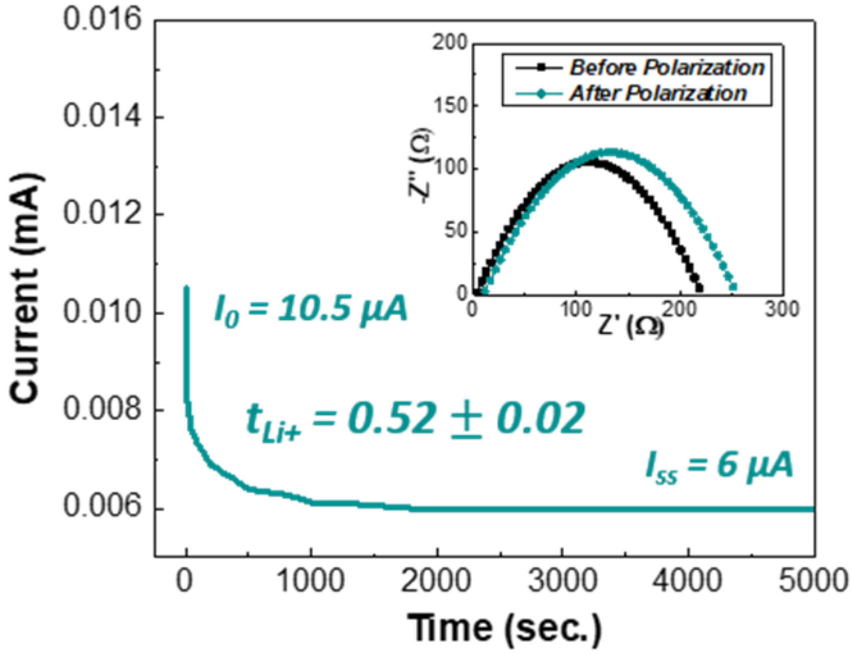

Figure S10. The chronoamperometry (CA) profile of the Li|PP|Li cell with a static potential of 10 mV. The corresponding EIS plots before and after the polarization are shown in the inset.

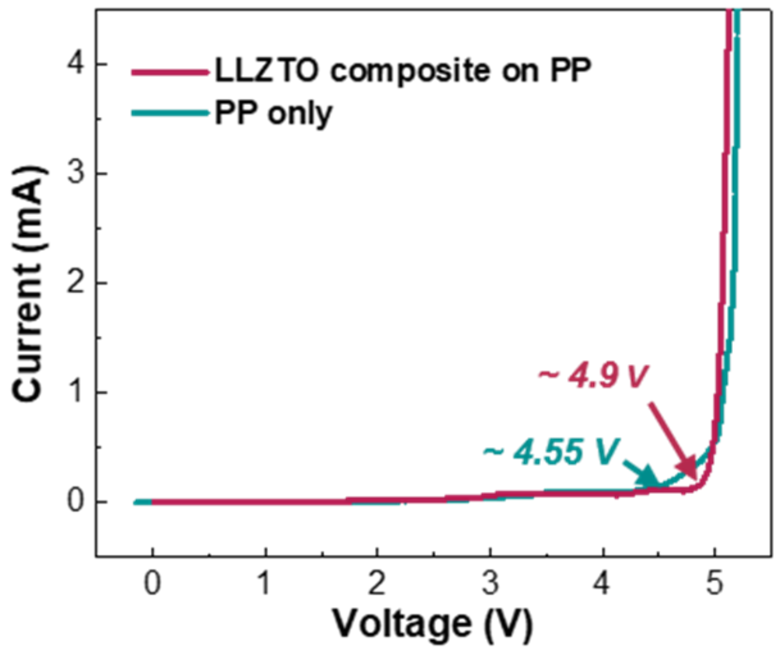

Figure S11. Linear sweep voltammetry (LSV) profiles of the PP separator and the SE/polymer composite on the PP separator.

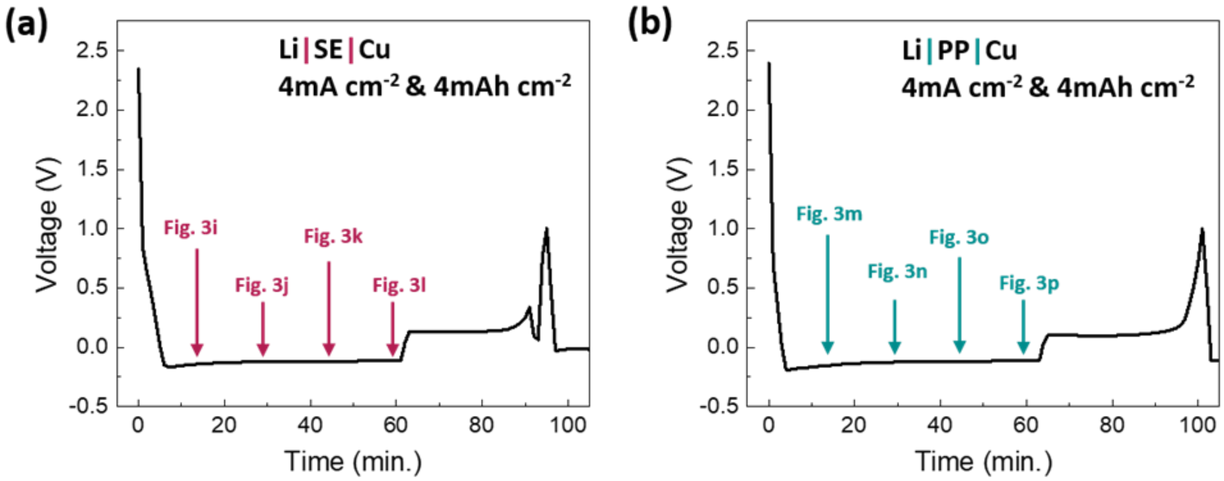

Figure S12. Li metal plating/stripping overpotential profiles for (a) Li|SE|Cu and (b) Li|PP|Cu asymmetric cells during the in-operando study at a current density of 4 mA cm^{-2} with a capacity of 4 mAh cm^{-2}.

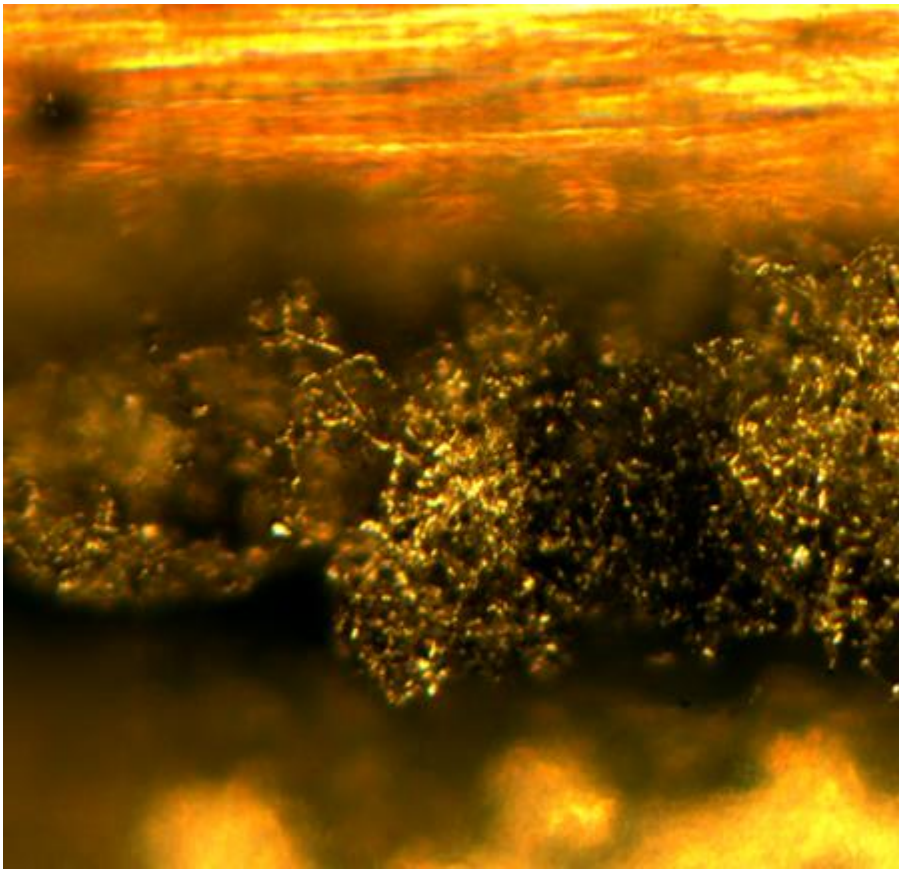

Figure S13. Li dendrites in focus for Fig. 4p.

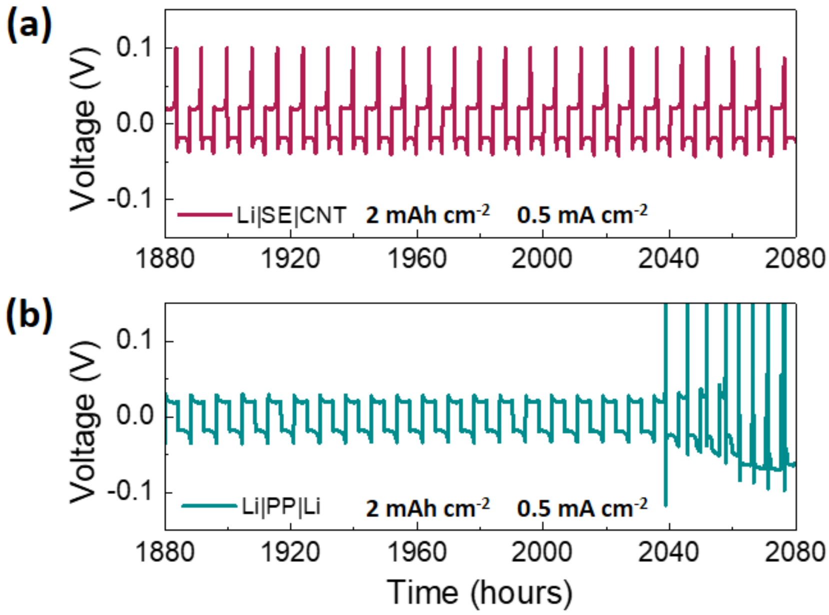

Figure S14. Li metal plating/stripping voltage profiles of (a) the Li|SE|CNT asymmetric cell and (b) the Li|PP|Li symmetric cell during 1880-2080 hours at a current density of 0.5 mA cm^{-2} with a capacity of 2.0 mAh cm^{-2}. Li|PP|Li failed after 2080 hours whereas Li|SE|CNT remained stable.

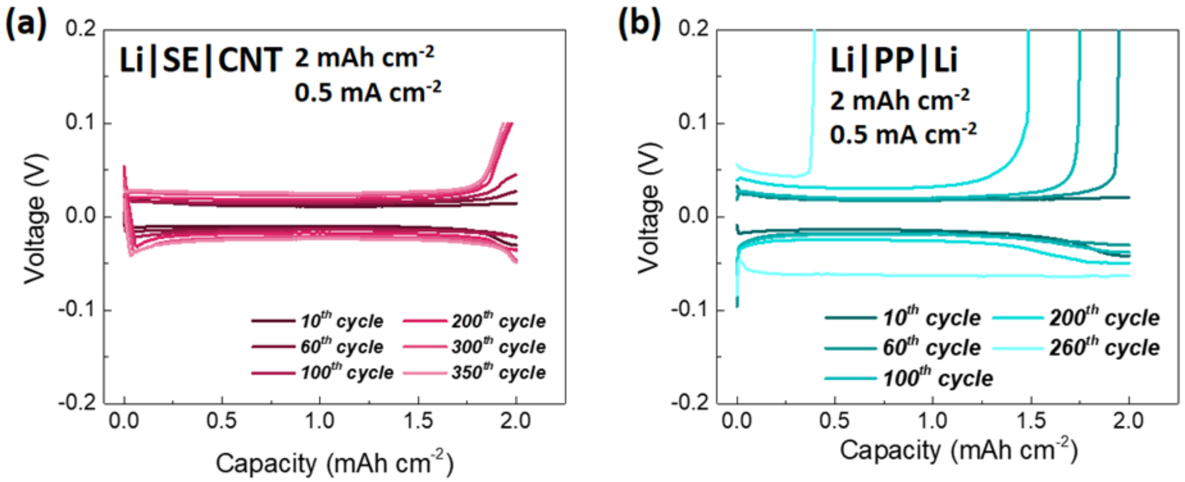

Figure S15. The voltage – capacity profiles under the Li metal plating/stripping cycling of (a) the Li|SE|CNT asymmetric cell at the 10th, 60th, 100th, 200th, 300th, and 350th cycles, and (b) the Li|PP|Li symmetric cell at the 10th, 60th, 100th, 200th, and 250th cycles.

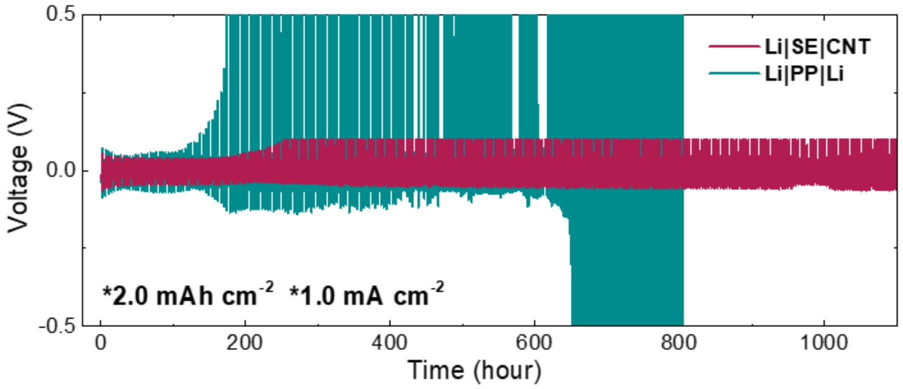

Figure S16. Voltage profiles of the Li|SE|CNT asymmetric cell (red) and the Li|PP|Li symmetric cell (green) at a current density of 1.0 mA cm^{-2} with a capacity of 2.0 mAh cm^{-2}

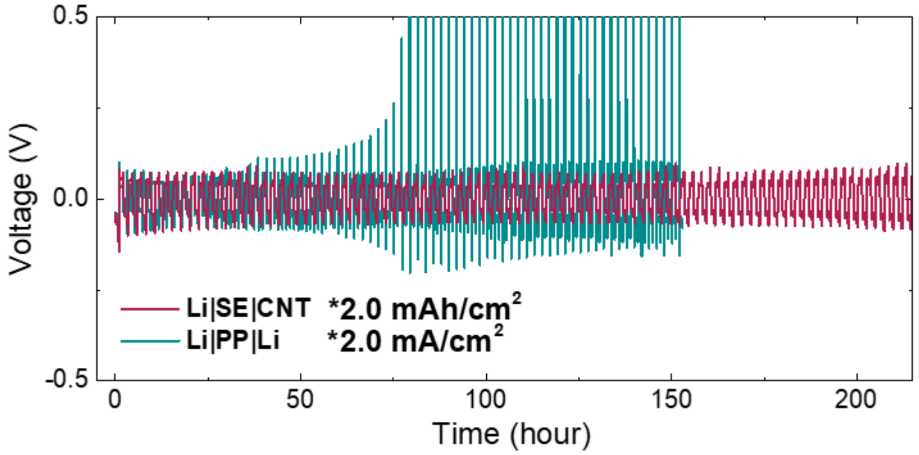

Figure S17. Voltage profiles of the Li|SE|CNT asymmetric cell (red) and the Li|PP|Li symmetric cell (green) at a current density of 2.0 mA cm^{-2} with a capacity of 2.0 mAh cm^{-2}

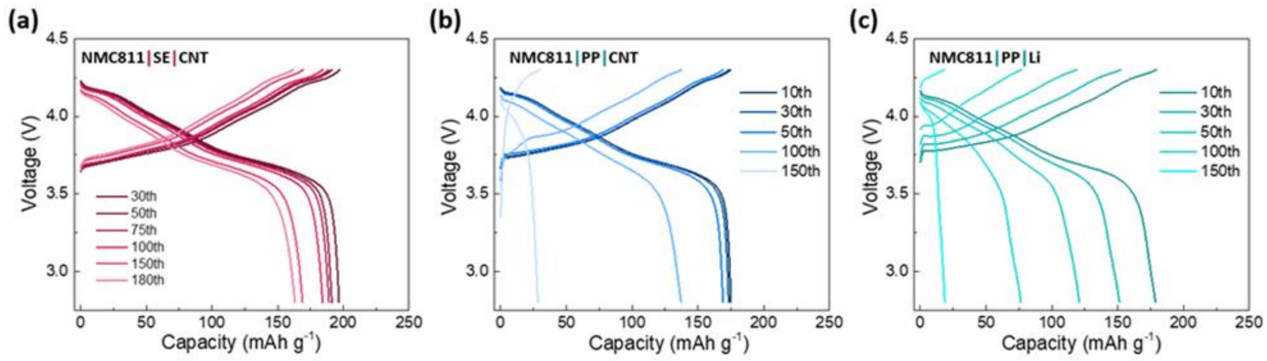

Figure S18. Voltage profiles of (a) the NMC811|SE|CNT cell at the 30th, 50th, 75th, 100th, 150th, and 180th cycles, (b) the NMC811|PP|CNT cell at the 10th, 30th, 50th, 100th, and 150th cycles, and (c) the NMC811|PP|Li full cell at the 10th, 30th, 50th, 100th, and 150th cycles with a voltage window of 2.8~4.3 V and a charging/discharging C-rate of 0.5 C.

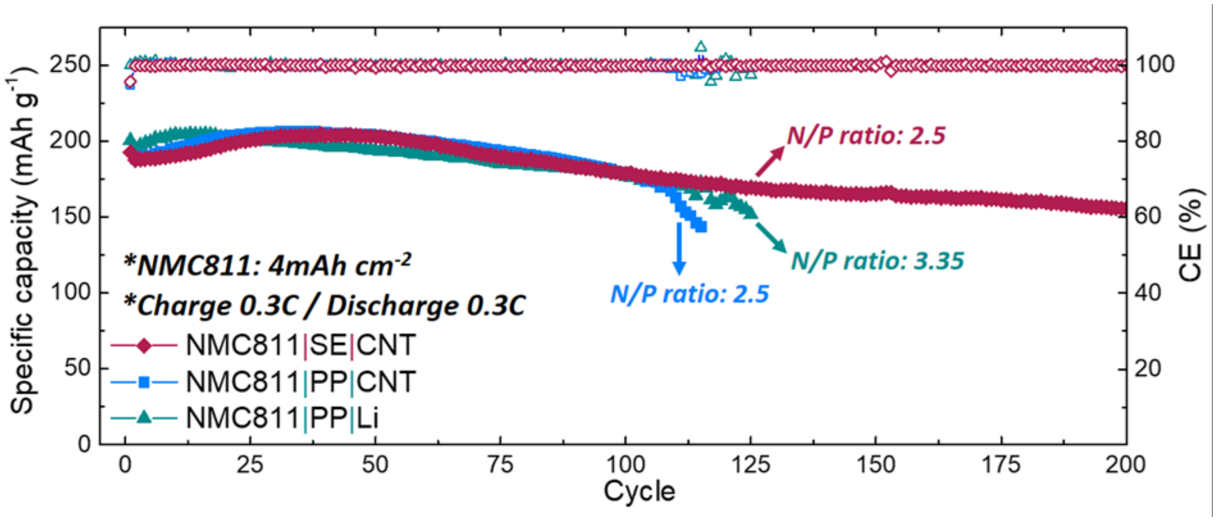

Figure S19. Discharge capacity (filled symbols) and Coulombic efficiency (hollow symbols) for the NMC811|SE|CNT, NMC811|PP|CNT, and NMC811|PP|Li full cells at a C-rate of 0.3 C with a voltage window of 2.8~4.3 V. The areal capacity of the NMC811 cathode is 4 mAh cm^{-2}, and the N/P ratios are 2.5 (NMC811|SE|CNT), 2.5 (NMC811|PP|CNT), and 3.4 (NMC811|PP|Li).

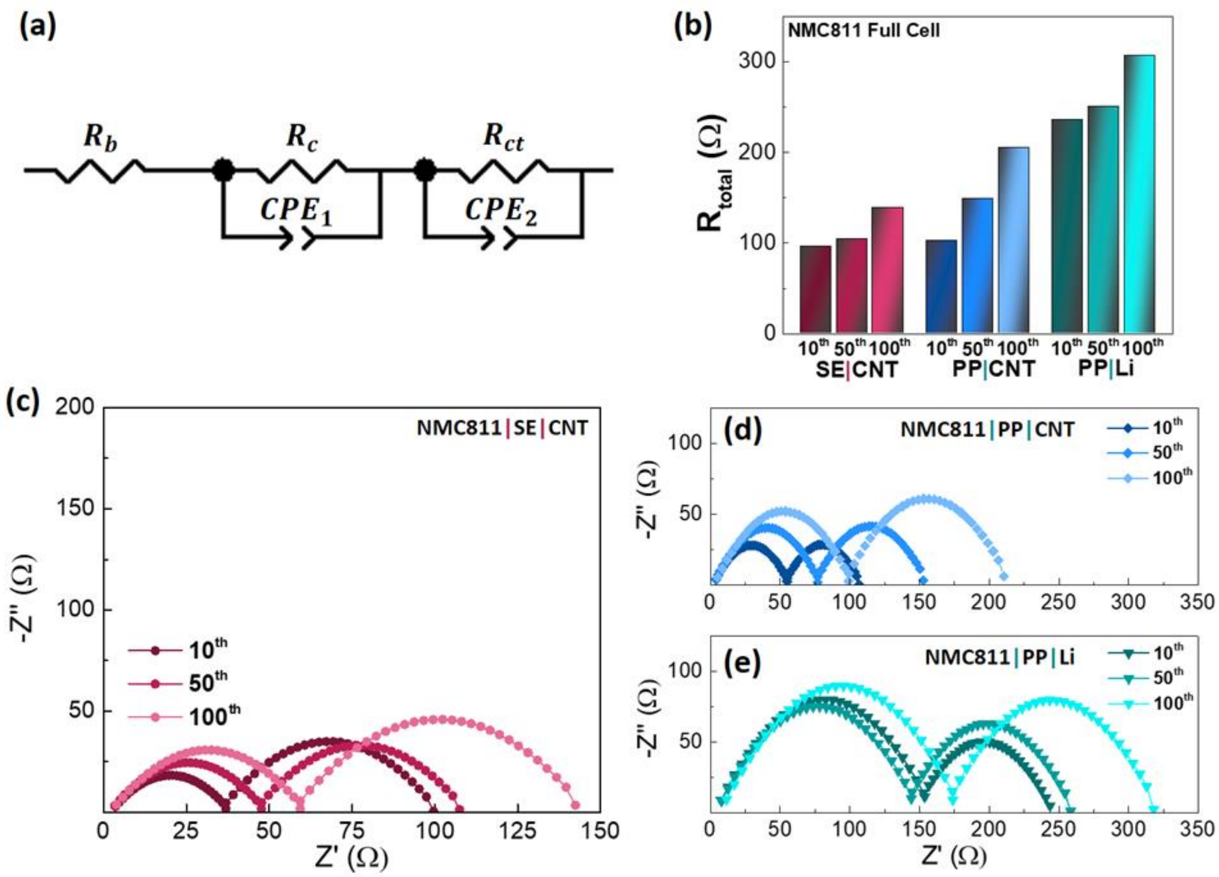

Figure S20. (a) The equivalent circuit for EIS data fitting for NMC811 full cells. The equivalent circuit consists of the bulk impedance (R_b), the interfacial contact impedance at the SEI layer (R_c), and the charge transfer impedance on the electrolyte/electrode interface (R_{ct}). CPE_1 and CPE_2 stand for the constant phase element. (b) The total impedance including R_b, R_c, and R_{ct}. (c-e) The EIS spectra of (c) NMC811|SE|CNT, (d) NMC811|PP|CNT, and (e) NMC811|PP|Li full cells at the fully discharged state at the10th, 50th, and 100th cycles.

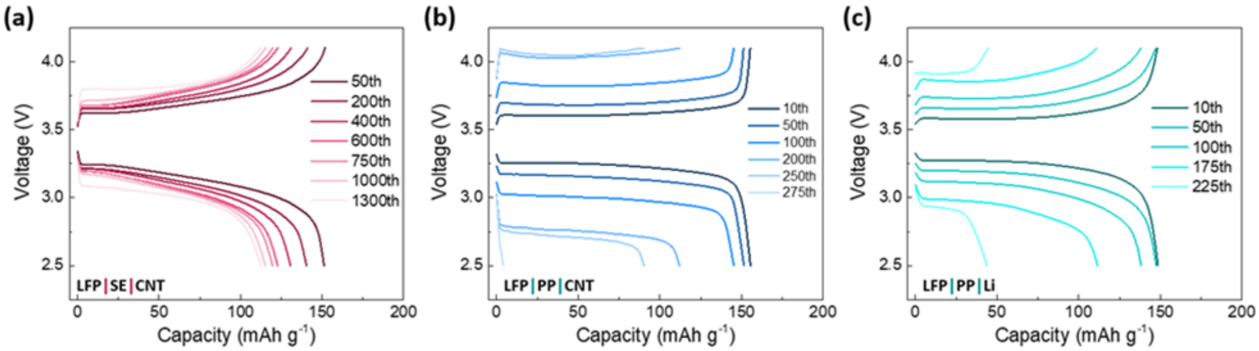

Figure S21. Voltage profiles of (a) the LFP|SE|CNT cell at the 50th, 100th, 200th, 300th, 400th, and 500th cycles, (b) the LFP|PP|CNT cell at the 10th, 50th, 100th, 200th, 250th, and 275th cycles, and (c) the LFP|PP|Li cell at the 10th, 50th, 100th, 175th, and 225th cycles with a voltage window of 2.5~4.1 V and a charging/discharging C-rate of 0.5.

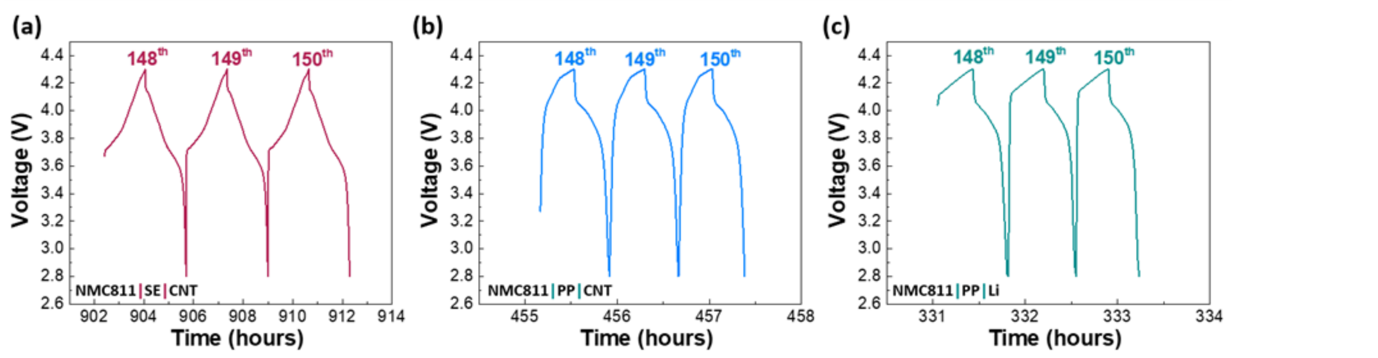

Figure S22. Charge/discharge voltage profiles of the NMC811 full cells with different separator|anode pairs near the 150th cycle - (a) NMC811|SE|CNT (b) NMC811|PP|CNT, and (c) NMC811|PP|Li.

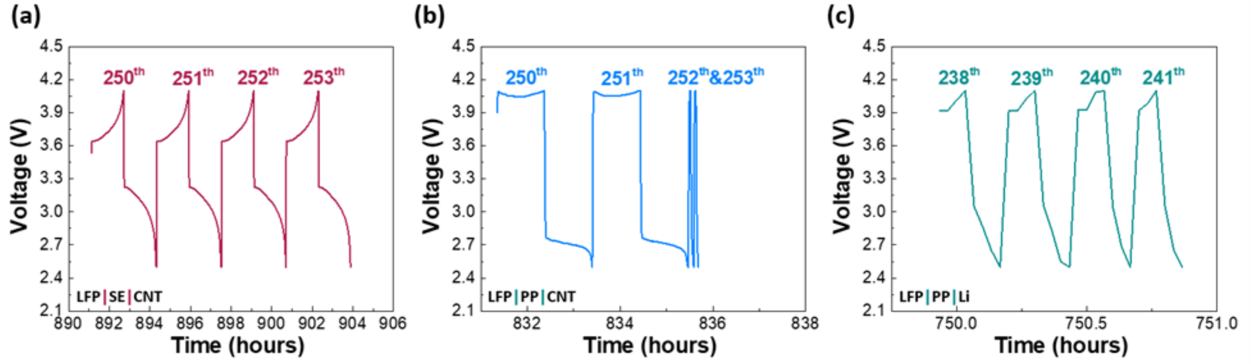

Figure S23. Charge/discharge voltage profiles of the LFP full cells with different separator|anode pairs near the 250th cycle - (a) LFP|SE|CNT, (b) LFP|PP|CNT, and (c) LFP|PP|Li.

Table S1. Standard deviation results of the Li^+ concentration beneath the separator for each separator/anode pair. Each calculation was conducted under 1 μm of the separator boundary.

Separator|Anode	PP|Li	SE|Li	PP|CNT	SE|CNT
Standard deviation of Li^+ concentration [M]	1.15 E-2	7.95 E-3	1.45 E-3	1.29 E-4

Table S2. Separator properties and ionic conductivity (σ).

Material	Thickness [μm]	Area [cm^2]	Bulk impedance (R_b) [Ω]	Ionic conductivity (σ) [mS cm^{-1}]
PP	25	1.89	2.45	0.54
SE15	45	1.58	2.48	1.15
SE35	45	1.44	2.05	1.53
SE50	45	1.32	2.00	1.70
SE75	45	1.99	2.76	0.82

Table S3. The average capacity values of the NMC811|SE|CNT and NMC811|PP|Li full cells.

Current density (Cycle)	Average discharge capacity of NMC811|SE|CNT (mAh g^{-1})	Average discharge capacity of NMC811|PP|Li (mAh g^{-1})
40 mA g^{-1} (1st – 5th)	202.6	204.1
60 mA g^{-1} (6th – 10th)	196.9	198.9
100 mA g^{-1} (11th – 15th)	186.6	178.0
200 mA g^{-1} (16th – 20th)	166.1	159.5
400 mA g^{-1} (21st – 25th)	142.3	120.3
200 mA g^{-1} (26th – 30th)	168.6	158.3
100 mA g^{-1} (31st – 35th)	185.2	184.8
60 mA g^{-1} (36th – 40th)	195.2	195.2
40 mA g^{-1} (41st – 45th)	201.0	200.3

Table S4. The average capacity values of the LFP|SE|CNT and LFP|PP|Li full cells.

Current density (Cycle)	Average discharge capacity of LFP|SE|CNT (mAh g^{-1})	Average discharge capacity of LFP|PP|Li (mAh g^{-1})
34 mA g^{-1} (1st – 5th)	162.8	161.0
51 mA g^{-1} (6th – 10th)	157.2	149.5
85 mA g^{-1} (11th – 15th)	148.4	136.1
170 mA g^{-1} (16th – 20th)	131.9	103.2
340 mA g^{-1} (21st – 25th)	82.3	59.7
170 mA g^{-1} (26th – 30th)	129.3	97.9
85 mA g^{-1} (31st – 35th)	144.0	125.0
51 mA g^{-1} (36th – 40th)	152.9	130.4
34 mA g^{-1} (41st – 45th)	157.0	156.6

Table S5. The bulk impedance data of the NMC811|SE|CNT, NMC811|PP|CNT, and NMC811|PP|Li full cells at the 10th, 50th, and 100th cycles.

Type / Cycle	R_b (Ω) (NMC811|SE|CNT)	R_b (Ω) (NMC811|PP|CNT)	R_b (Ω) (NMC811|PP|Li)
10th	3.4	3.8	7.5
50th	3.3	3.9	7.9
100th	3.8	5.2	11.5

Table S6. The interfacial contact impedance data of the NMC811|SE|CNT, NMC811|PP|CNT, and NMC811|PP|Li full cells at the 10th, 50th, and 100th cycles.

Type / Cycle	R_c (Ω) (NMC811|SE|CNT)	R_c (Ω) (NMC811|PP|CNT)	R_c (Ω) (NMC811|PP|Li)
10th	33.4	51.1	146.1
50th	44.3	72.9	136.1
100th	55.5	93.9	162.2

Table S7. The charge-transfer impedance data of the NMC811|SE|CNT, NMC811|PP|CNT, and NMC811|PP|Li full cells at the 10th, 50th, and 100th cycles.

Type / Cycle	R_{ct} (Ω) (NMC811|SE|CNT)	R_{ct} (Ω) (NMC811|PP|CNT)	R_{ct} (Ω) (NMC811|PP|Li)
10th	62.7	51.7	89.9
50th	59.9	75.8	114.2
100th	83.2	111.2	144.0

Table S8. Detailed information of the commercial Li-ion batteries in Fig. 8b[S15–S18] along with our NMC811|SE|CNT full cell under the lean electrolyte condition. The 5th column describes the specific energy density values in the literature.[S18] The 6th column represents the calculated specific energy density values excluding the external housing/case. In the rightmost column, the number in the bracket is the initial production year.

**Label**	**Catho-de**	**Anode**	**Dis-charge voltage (V)**	**Gravimetric energy density (literature data) (Wh kg^{-1})**	**Gravimetric energy density excluding housing/case (Wh kg^{-1})**	**Volumetric energy density (Wh L^{-1})**	**Manufacturer /Model (Year)**
This work	NMC	CNT	3.85	-	**334**	**783**	-
LG1	NMC	Graphite	3.65	241	**290**	**466**	LG chem./ Renault Zoe (2017)
LG2	NMC	Graphite	3.7	186	**224**	**393**	LG chem./ Chevrolet Bolt (2016)
LT	NMC	Graphite	3.65	152	**183**	**316**	Li-Tec/ Smart Fortwo EV (2013)
PS	NMC	Graphite	3.7	130	**157**	**215**	Panasonic-Sanyo/ VW e-Golf (2015)
S	LMO-NMC	Graphite	3.65	172	**207**	**312**	Samsung SDI/ Fiat 500e (2013)
LG3	LMO-NMC	Graphite	3.75	157	**189**	**275**	LG chem./ Renault Zoe (2012)
J	LMO-NMC	Graphite	3.7	109	**131**	**218**	Li Energy Japan/ Mitsubishi i-MIEV (2008)

References

S1. Yang, G.; Choi, W.; Pu, X.; Yu, C., Scalable synthesis of bi-functional high-performance carbon nanotube sponge catalysts and electrodes with optimum C–N–Fe coordination for oxygen reduction reaction. *Energy & Environmental Science* **2015,** *8* (6), 1799-1807.

S2. Yang, G.; Tan, J.; Jin, H.; Kim, Y. H.; Yang, X.; Son, D. H.; Ahn, S.; Zhou, H.; Yu, C., Creating effective nanoreactors on carbon nanotubes with mechanochemical treatments for high-areal-capacity sulfur cathodes and lithium anodes. *Advanced Functional Materials* **2018,** *28* (32), 1800595.

S3. Noh, J.; Tan, J.; Yadav, D. R.; Wu, P.; Xie, K. Y.;Yu, C., Understanding of lithium insertion into 3d porous carbon scaffolds with hybridized lithiophobic and lithiophilic surfaces by in-operando study. *Nano Letters* **2020,** *20* (5), 3681-3687.

S4. A Shiraishi, S., Kanamura, K. & Takehara, Zi Surface condition changes in lithium metal deposited in nonaqueous electrolyte containing HF by dissolution-deposition cycles. J. Electrochem. Soc. 146, 1633–1639 (1999).

S5. Mogi, R.; Inaba, M.; Jeong, S.-K.; Iriyama, Y.; Abe, T.; Ogumi, Z., Effects of some organic additives on lithium deposition in propylene carbonate. *Journal of The Electrochemical Society* **2002,** *149* (12), A1578.

S6. Zheng, J.; Engelhard, M. H.; Mei, D.; Jiao, S.; Polzin, B. J.; Zhang, J.-G.; Xu, W., Electrolyte additive enabled fast charging and stable cycling lithium metal batteries. *Nature Energy* **2017,** *2* (3), 1-8.

S7. Xia, L.; Lee, S.; Jiang, Y.; Xia, Y.; Chen, G. Z.; Liu, Z., Fluorinated electrolytes for li-ion batteries: The lithium difluoro (oxalato) borate additive for stabilizing the solid electrolyte interphase. *ACS omega* **2017,** *2* (12), 8741-8750.

S8. Adams, B. D.; Zheng, J.; Ren, X.; Xu, W.; Zhang, J. G., Accurate determination of coulombic efficiency for lithium metal anodes and lithium metal batteries. *Advanced Energy Materials* **2018,** *8* (7), 1702097.

S9. Hwang, J.-Y.; Park, S.-J.; Yoon, C. S.; Sun, Y.-K., Customizing a Li–metal battery that survives practical operating conditions for electric vehicle applications. *Energy & Environmental Science* **2019,** *12* (7), 2174-2184.

S10. Sun, H.-H.; Dolocan, A.; Weeks, J. A.; Rodriguez, R.; Heller, A.; Mullins, C. B., In situ formation of a multicomponent inorganic-rich SEI layer provides a fast charging and high specific energy Li-metal battery. *Journal of Materials Chemistry A* **2019,** *7* (30), 17782-17789.

S11. Ren, X.; Zou, L.; Cao, X.; Engelhard, M. H.; Liu, W.; Burton, S. D.; Lee, H.; Niu, C.; Matthews, B. E.; Zhu, Z., Enabling high-voltage lithium-metal batteries under practical conditions. *Joule* **2019,** *3* (7), 1662-1676.

S12. Rudyak, V. Y.; Tretiakov, D. S., On diffusion of single-walled carbon nanotubes. *Thermophysics and Aeromechanics* **2020,** *27* (6), 847-855.

S13. Chen, R.; Qu, W.; Guo, X.; Li, L.; Wu, F., The pursuit of solid-state electrolytes for lithium batteries: from comprehensive insight to emerging horizons. *Materials Horizons* **2016,** *3* (6), 487-516.

S14. Scrosati, B.; Garche, J.; Tillmetz, W., *Advances in battery technologies for electric vehicles*. Woodhead Publishing: 2015.

S15. Blomgren, G. E., The development and future of lithium ion batteries. *Journal of The Electrochemical Society* **2016,** *164* (1), A5019.

S16. Anderman, M. In *xEV Expansion, Key Technology, and Market Development*, 2017.

S17. Schmuch, R.; Wagner, R.; Hörpel, G.; Placke, T.;Winter, M., Performance and cost of materials for lithium-based rechargeable automotive batteries. *Nature Energy* **2018,** *3* (4), 267-278.

S18. Geldasa, F. T.; Kebede, M. A.; Shura, M. W.;Hone, F. G., Identifying surface degradation, mechanical failure, and thermal instability phenomena of high energy density Ni-rich NCM cathode materials for lithium-ion batteries: a review. *RSC Advances* **2022,** *12* (10), 5891-5909.

S19. Steinhart, M.; Senz, S.; Wehrspohn, R. B.; Gösele, U.;Wendorff, J. H., Curvature-directed crystallization of poly (vinylidene difluoride) in nanotube walls. *Macromolecules* **2003,** *36* (10), 3646-3651.

S20. Shi, X.; Sun, Q.; Boateng, B.; Niu, Y.; Han, Y.; Lv, W.; He, W., A quasi-solid composite separator with high ductility for safe and high-performance lithium-ion batteries. *Journal of Power Sources* **2019,** *414*, 225-232.

S21. Ali, S.; Tan, C.; Waqas, M.; Lv, W.; Wei, Z.; Wu, S.; Boateng, B.; Liu, J.; Ahmed, J.; Xiong, J., Highly efficient PVDF-HFP/colloidal alumina composite separator for high-temperature lithium-ion batteries. *Advanced Materials Interfaces* **2018,** *5* (5), 1701147.

S22. Ding, Y.; Zhang, P.; Long, Z.; Jiang, Y.; Xu, F.; Di, W., The ionic conductivity and mechanical property of electrospun P (VdF-HFP)/PMMA membranes for lithium ion batteries. *Journal of membrane science* **2009,** *329* (1-2), 56-59.

S23. Gregorio, J. R.; Cestari, M., Effect of crystallization temperature on the crystalline phase content and morphology of poly (vinylidene fluoride). *Journal of Polymer Science Part B: Polymer Physics* **1994,** *32* (5), 859-870.
